# Development and Optimisation of a Multiresidue Method for the Determination of 40 Anthelmintic Compounds in Environmental Water Samples by Solid Phase Extraction (SPE) with LC-MS/MS Detection

**DOI:** 10.3390/molecules24101978

**Published:** 2019-05-22

**Authors:** Damien Mooney, Catherine Coxon, Karl G Richards, Laurence Gill, Per-Erik Mellander, Martin Danaher

**Affiliations:** 1 School of Natural Sciences, Geology Department, Trinity College Dublin, D02PN40 Dublin, Ireland; cecoxon@tcd.ie; 2 Food Safety Department, Teagasc Food Research Centre, Ashtown, D15KN3K Dublin 15, Ireland; Martin.Danaher@teagasc.ie; 3 Groundwater spoke, Irish Centre for Research in Applied Geosciences (iCRAG), D04N2E5 Dublin, Ireland; Karl.Richards@teagasc.ie (K.G.R.); Laurence.Gill@tcd.ie (L.G.); 4 Environment, Soils and Land-Use Department, Environment Research Centre, Teagasc, Johnstown Castle, Y35TC97 Wexford, Ireland; PerErik.Mellander@teagasc.ie; 5 Department of Civil, Structural and Environmental Engineering, Trinity College Dublin, D02PN40 Dublin, Ireland

**Keywords:** veterinary drugs, anthelmintics, emerging organic contaminants, transformation products, environmental water, solid phase extraction, UHPLC-MS/MS

## Abstract

A comprehensive multiresidue method was developed and validated for the determination of 40 anthelmintic compounds, including 13 transformation products, in surface and groundwater samples at sub nanogram per litre (ng L^−1^) levels. Anthelmintic residues were extracted from unfiltered water samples using polymeric divinylbenzene solid phase extraction (SPE) cartridges, and eluted with methanol: acetone (50:50, *v*/*v*). Purified extracts were concentrated, filtered and injected for UHPLC-MS/MS determination. The method recovery (at a concentration representative of realistic expected environmental water levels based on literature review) ranged from 83–113%. The method was validated, at three concentration levels, in accordance to Commission Decision 2002/657/EC and SANTE/11813/2017 guidelines. Trueness and precision, under within-laboratory reproducibility conditions, ranged from 88–114% and 1.1–19.4%, respectively. The applicability of the method was assessed in a pilot study whereby 72 different surface and groundwater samples were collected and analysed for the determination of these 40 compounds for the first time in Ireland. This is the most comprehensive method available for the investigation of the occurrence of both anthelmintic parent compounds and their transformation products in raw, unfiltered environmental waters.

## 1. Introduction

Due to increased pressures on the food production system, veterinary antiparasitic agents, such as anthelmintic drugs, have become a critical component of animal husbandry in many countries, including Ireland. Anthelmintic drugs are widely used to control helminthic parasites that infect animals, particularly those exposed through pasture-based production systems. Anthelmintics are primarily used to treat and prevent the following parasitic worms in ruminants: nematodes, cestodes and trematodes, which are more commonly known as roundworms, tapeworms and liver flukes, respectively [1]. The anthelmintic family can be divided into a number of groups or classes, primarily based on their chemical structure, and their mode of action against the parasite [2]. The main classes of anthelmintics include: the benzimidazoles, macrocyclic lactones (avermectins and milbemycins), salicylanilides and substituted phenols, tetrahydropyrimidines, imidazothiazoles, organophosphates and amino-acetonitrile derivatives. The compounds included in this study, as grouped into their respective anthelmintic class, are listed in Table 1, with their structures shown in Supplementary Information Figure S1. 

Anthelmintics can be administered orally (drench or bolus), as an injectable preparation or topically (pour-on). Once administered, the drug can undergo a series of transformations within the animal, eventually being excreted as the parent drug and/or metabolites in urine or faeces [3,4,5], the exact excretion profile of which is equally dependent on the route of administration and the drug’s physicochemical properties [6]. As a result, the excretion data available for antiparasitic agents is limited and often difficult to interpret or compare [5]. However, of the information available, it has been shown that >90% of the administered dose of avermectins can be excreted in faeces as the unchanged parent [7,8,9], while in contrast, the benzimidazoles, levamisole and tetrahydropyrimidines are mainly excreted in urine as parent and /or metabolites [10]. As a result, it is evident that the administration of such ‘agrochemicals’ can potentially lead to their persistence in the environment, posing a risk to water quality, which has led to anthelmintics being considered as emerging organic contaminants (EOCs) of potential concern [11,12,13].

The most important point of entry for anthelmintics into the environment is due to the direct excretion onto pastures and/ or by direct application of slurries to land [14,15]. Boxall et al. [16] emphasised the importance of wash-off of topical treatments and spillage during application as other important routes to enter the environment. Once in the environment, the fate and transport of anthelmintic drugs is further complicated due to their breakdown into transformation products (TPs), which can be more toxic than the parent drug [16,17]. The ecotoxicity of anthelmintic drugs in the environment is not well established; however, some of these drugs have been found to be toxic to different organisms in the environment, as summarised in a recent review by Horvat et al. [13]. For example, the avermectins, as a group, have been found to have effects on the reproduction, biological function and survival of nontarget terrestrial and aquatic organisms. For instance, ivermectin is acutely toxic to crustaceans, with an LC_50_ of low ng L^−1^ levels [9]. O’Hea et al. [18] also highlighted the impact of ivermectin in the environment on dung beetle populations.

While there are well defined legislative requirements pertaining to veterinary residues of anthelmintic drugs in food of animal origin, there is no specific legislation relating to emerging organic contaminants in environmental waters. Environmental marker residues are not well defined in legislation in contrast to those listed under food safety legislation. This is due to the very nature of EOCs, since often there is very little information known about the fate and toxicity of such substances. There are some individual pieces of legislation relating to pesticides in environmental waters in the European Union (EU), such as the drinking water Directive 98/83/EC [19] and groundwater Directive 2006/118/EC [20]. Under such legislation, the term ‘nematocides’ is included under the definition of pesticide. As a result, the individual and total pesticide limits (100 ng L^−1^ and 500 ng L^−1^ respectively) specified, are applicable to some anthelmintic drugs. 

Liquid chromatography tandem mass spectrometry (LC-MS/MS) is currently considered the most powerful technique for the quantitative determination of a large number of veterinary residues in complex matrices [21]. Advances in detection systems have allowed for the development of multiclass methods for determining pharmaceuticals and veterinary drugs in environmental samples. Detection methods for environmental samples are not as well established (generally <40 analytes) [22,23], compared to those for biological matrices (hundreds of analytes) [24,25]. Methods for the LC-MS/MS detection of anthelmintic in environmental water samples are not very extensive and only include a limited number of analytes from the one class (generally <12 residues) [23,26,27,28]. Methodologies incorporating transformation products are scarce, with most method covering just parent drugs and not metabolites/environmental TPs (only four or fewer metabolites/TPs included in any one method, all of which relate to fenbendazole [22,26,29]). More extensive and sensitive methods have been developed for biological food matrices, which allow simultaneous detection of many more anthelmintic residues [30,31].

Regardless of the instrumental technique, due to the wide range of physicochemical properties of analytes and the complexity of environmental matrices, sample preparation steps are inevitable in order to achieve the required sensitivity. A number of different extraction and purification techniques have been applied for the determination of anthelmintic drugs, with solid phase extraction (SPE) being the most commonly used technique when it comes to environmental water samples. Of the available extraction methods specific to water matrices, the majority are considered multiresidue methods incorporating more than one anthelmintic; however, in most cases, these methods are limited to <10 anthelmintic compounds [26,27,28], or incorporate only 1–2 anthelmintics, as part of multiclass determinations of various pharmaceuticals [23,32]. The most comprehensive method, to our knowledge, was that developed by Zrncic et al. [26] who proposed a method for the multiresidue determination of ten anthelmintics from differing structural classes (the majority being from the benzimidazole class), from surface river water using SPE (HLB cartridge; 60 mg, 3 mL). Krogh et al. [27] presented a method for the extraction and determination of seven avermectins from surface water (500 mL), also using polymeric SPE; however, recoveries reported were relatively low (38–67%). 

In a prioritisation exercise on veterinary medicines in the environment in the United Kingdom (UK), Boxall et al. [17] identified 56 different drugs, including eight anthelmintics, which they considered to be of high priority with regards to having a potential impact on the environment. The same working group also noted the lack of suitably sensitive analytical methodology, specifically for TPs, as one of the main contributors to inadequate environmental risk assessment [6,16]. Even though there are some methods available for a limited number of anthelmintics, information on the occurrence and associated levels in the environment is lacking. Information on the occurrence of anthelmintic metabolites and transformation products is even more scarce [13], which further hinders sufficient environmental risk assessment. Some studies even go as far as questioning whether current legislation and environmental risk assessments of both human and veterinary products are sufficiently protective [33]. In order to better inform on the environmental fate and occurrence of anthelmintics in environmental waters, more comprehensive analytical methods capable of detecting many more anthelmintics and their TPs, at environmentally relevant detection levels, are required. The objective of this study therefore was to develop, optimise and validate a more comprehensive method for the multiresidue determination of a wide range of commonly used anthelmintics and their transformation products, incorporating clean-up by SPE. This method was then applied to a wide range of surface and groundwater samples from across Ireland, to help better understand the environmental fate and occurrence of anthelmintics. 

## 2. Results and Discussion

### 2.1. Method Development: Sample Preparation—Solid Phase Extraction 

#### 2.1.1. Assessment of SPE Sorbents 

**Figure 1:**
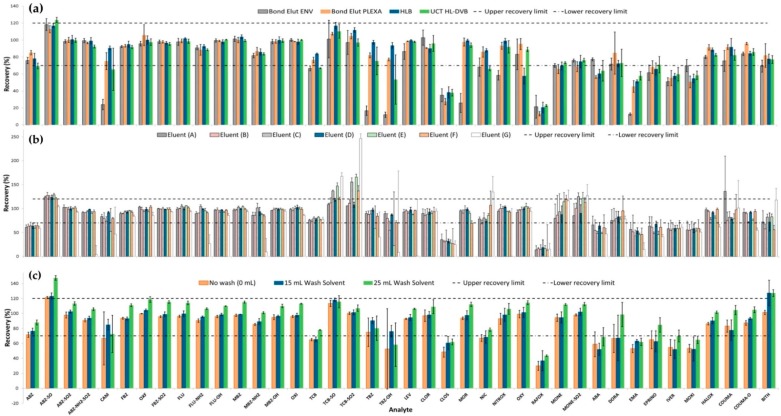
Mean recoveries (%) and precision (%RSD, shown by error bars)(*n* = 3) for assessment of: (**a**) four different SPE Cartridges (Bond Elut ENV, Bond Elut Plexa, Oasis HLB and UCT HL-DVB) eluted with 50: 50 MeOH: MeCN (v/v) (10 mL) (**b**) seven different eluent compositions: (A) = 50/50 MeOH/MeCN (10 mL), (B) = 50/50 MeOH/MeCN (5 mL) + Acetone (5 mL), (C) = 50/50 MeOH/MeCN (5 mL) + MTBE (5 mL) (D) = 50/50 MeOH/Acetone (10 mL), (E) = 50/50 MeCN/MTBE (10 mL), (F) = 100% Acetone (10 mL) and (G) = 100% MTBE (10 mL) using the HL-DVB cartridge (200 mg, 6 mL) and (**c**) three different volumes (0, 15 and 25 mL) of water: methanol (90:10, v/v) wash solution using the same HL-DVB cartridge.

Amongst the literature, polymeric hydrophilic-lipophilic type sorbents are most commonly used for the SPE of anthelmintic drugs from water, due to their all-purpose, strong hydrophilic reversed phase application for the extraction of pharmaceuticals [22,26,27,28]. As a result, method development and optimisation tasks focused on the use of such sorbents, with initial experiments focusing on the use of Bond Elut ENV reversed phase polymeric cartridges, which have large particle size for high volume, fast flow-through application. Investigation of elution solvent composition ((methanol (MeOH)/ acetonitrile (MeCN)) and volume (0–15 mL) indicated optimum conditions with a MeOH: MeCN (50:50, *v*/*v*), 10 mL, elution (data not shown). However, no further optimisation of this particular sorbent was carried out, due to inconsistencies with the SPE sorbent packing after vacuum drying, which produced large deviations in recoveries between replicates (RSD >30%). Using the same elution conditions optimised for the Bond Elut ENV cartridge above, three additional SPE cartridges (Bond Elut PLEXA, Oasis HLB, and UCT HL-DVB) were assessed for extraction, with the results as depicted in Figure 1a. Oasis HLB performed best in terms of recoveries and precision, with 31 of 40 compounds extracted within the satisfactory recovery range and RSD values between 0.8–9.9%. UCT HL-DVB also achieved satisfactory recoveries for 31 of the 40 anthelmintics however for a few analytes the precision (%RSD) was larger (0.4–24.9%). Recovery of CLOS and RAFOX (<40 and <20% respectively) from all four sorbents were much lower than the minimum targeted recovery of 70%. Both these analytes have high octanol-water coefficients (K_ow_) (Supplementary Table S1), which implies that they are highly hydrophobic, thus the low recoveries were proposed to be due to inefficient elution from the sorbents, or adsorption of these analytes on the sample container wall. The UCT HL-DVB was selected for further assessment due to its faster sample load times compared to HLB and PLEXA, which both required much higher vacuum, increasing the load time by 60 min. 

Further to this, sorbent mass (200 mg vs. 500 mg) and elution volume (10, 15 and 20 mL), were assessed for the HL-DVB cartridge, with the 200 mg cartridge combined with a 10 mL elution volume providing the best result (Supplementary Figure S2). CAM, TBZ and TBZ-OH all showed no extraction (all < 0.5% extraction) with the 500 mg sorbent mass; therefore, 200 mg was selected. This is most likely due to too much retention on the larger bed mass. The 10 mL elution volume was selected given there was no noticeable difference observed on increasing the volumes, in addition to the fact that larger volumes were restricted by evaporation capabilities (max. 15 mL tube in TurboVap LV). The selected elution volume was similar to those reported amongst other published methods [23,27,32]. 

#### 2.1.2. Elution Solvent Composition and Wash Solvent Assessment

Optimisation of elution solvent composition for the UCT HL-DVB 200 mg cartridge was performed given that increases in the eluent volumes (Section 2.1.1) did not improve recoveries. Seven different elution solvent compositions, (A)–(G), were assessed, with the mean recoveries and RSDs (n = 3) obtained for each composition presented in Figure 1b. These compositions were selected based on commonly used elution solvents for these compounds in the literature [22,28,32]. The best results were determined to be with elution with composition (D) which produced recoveries in the range of 19–123% and precision of 0.5–18.8%. Eluent (A) produced similar recoveries (14–136%); however, (D) was preferred as it produced more precise results across replicates (improved RSD for ABA, CLOS, COUMA, DORA, EMA, EPRINO, IVER and MOXI), with increased sensitivity also observed (higher analyte responses). This increased sensitivity was proposed to be due to less polar interferences being eluted by the more hydrophobic acetone solvent incorporated in Eluent (D) (compared to MeCN in (A)). There was still no significant improvement for CLOS and RAFOX indicating the lower recoveries may not be due to insufficient elution from the sorbent. 

The effect of a mild wash solvent (90:10 (*v*/*v*) H_2_O: MeOH) was assessed in order to remove undesirable matrix coextractives from the SPE, prior to analyte elution. The use of no wash solvent was compared to the use of 15 mL or 25 mL wash aliquots (used to rinse the sample container, before being added to the SPE). The best results were achieved with use of the 15 mL wash solution (recoveries of 37–127%), with improved recoveries observed for all analytes, except ABA, IVER and COUMA, which had slightly decreased recoveries compared to no wash step (Figure 1c). While the avermectins showed improved recoveries when the larger wash aliquot (25 mL) was incorporated (recoveries increased by up to 33%), lower recoveries and poorer precision were demonstrated for a number of other analytes (e.g., CAM, TBZ and TBZ-OH). With both wash volumes, the recovery of CLOS and RAFOX increased by at least 10%, most likely due to more efficient extraction of analytes that may have remained adsorbed to the glass surface of the sample container.

#### 2.1.3. Sample Modification (Organic Modifier and pH)

Sample modification was assessed to further investigate low recoveries of some analytes due to potential inefficient extraction of analyte from the sample, and its container. Thompson et al. [34] report that the addition of MeOH to samples was necessary to prevent partial adsorption of analytes (which included avermectins) to container surfaces; thus, the use of a methanol modifier was assessed in this study. Krogh et al. [27] report that sample pH did not have a drastic effect on the extraction of seven avermectins using HLB SPE; however, Zrncic et al. [26] indicated that pH can affect the recovery of anthelmintics from other structural classes. As a result, the effect of sample pH modification was also assessed.

The best overall conditions from the response surface methodology (RSM) optimiser, for 17 selected compounds (see Section 3.6.1 below), were predicted to be extraction with 20% MeOH modifier at sample pH 7 (Supplementary Figure S3a). There was no notable change in predicted recoveries using 20 to 25% MeOH modifier; however, on further increasing the modifier (to 30%), the recoveries of a number of analytes greatly reduced (e.g., ABZ-SO, FBZ, TBZ-OH, LEV, CLOR and NITROX). In contrast, as the modifier is increased, the predicted recovery of CAM and a number of avermectins (EMA, EPRINO and MOXI) all increased, which is consistent with the necessity of organic modifier, as reported by Thompson et al. [34]. For sample pH, the optimum was predicted to be pH 7, with predicted recoveries of the majority of analytes consistent across the pH range of 6–8. At low pH (towards pH 4), recoveries were improved for a number of analytes, mainly belonging to the benzimidazole class (e.g., ABZ-SO, FBZ). This is due to the drugs becoming more ionised and more solubilised at lower pH, as a result of their first dissociation constant (pKa) being between 2.5–5.5 (Supplementary Table S1). The avermectins are neutral compounds, except EMA which is a salt and favours increased retention as the pH increases from 4 to 7, where the benzoate form will be prominent (pKa 4.7) and the epi methyl-amino ion will be almost 50% ionised (pKa 7.7) At basic pH, for a number of compounds, the recoveries predicted are lower compared to those at neutral and acidic pH, with the exception of CAM, FLU-NH_2_, LEV and MBZ-NH_2_, which all have basic functional groups and therefore will be less ionised and retained better at higher pH. These results are similar to that observed by Zrncic et al. [26] who assessed the effect of pH on the extraction of 10 anthelmintics from river water. At pH 4.0 these authors report the recovery of all analytes to be >60% with the exception of LEV (<20%) and MOXI (approx. 40%); however, on further increasing pH from acidic to neutral (pH 7.0), recoveries for the majority of the analytes further increased or remained the same. At basic pH, the authors report that the recovery of most analytes significantly decreased; however, the recovery of LEV was at its highest (>55%), as was predicted for LEV by the RSM optimiser graph in this work. In this current work, the overall response surface methodology predicted extraction pH range of 6–8 is consistent with the findings of Zrncic et al. (final pH 7 selected) and with other methods reported amongst literature [23,27,34]. The RSM graphs for the remaining 23 anthelmintics (Supplementary Figure S3b) showed that the predicted optimum modifier (20%) and pH conditions (pH 7) also gave satisfactory predicted recoveries.

The predicted results for sample modifier (%) were verified by application to fortified groundwater samples (*n* = 3) in which the average recoveries of analytes in samples using the optimal conditions (20% modifier, pH 7) were compared to the average recoveries in fortified samples without modifier addition (0% modifier, pH 7) (results depicted in Supplementary Figure S4). Ten of the 40 compounds showed an increase in recovery with the addition of the 20% modifier, while three compounds had a notable decrease in recovery. Notably, for the first time, acceptable recoveries for CLOR and RAFOX were verified with the incorportion of the modifier (recoveries of 91 and 75% with modifier compared to 50 and 28% with no modifier). Levamisole (LEV) showed the greatest decrease in recovery with the addition of the modifier (reduced from 89 to 70%), which indicates that the MeOH modifier causes breakthrough of LEV while loading; however, this recovery was still acceptable.

### 2.2. Method Validation

The method was validated at three concentration levels according to an amalgamation of criteria as specified in Table 1 (see Table 2 for validated concentration levels). 

#### 2.2.1. Identification

For each compound, one precursor and two daughter ions (one quantifier and one qualifier) were monitored, giving a total of four identification points, satisfying the confirmation criteria. Daughter ions were identified as part of the initial tuning of analytes on the MS detection system, with quantifier and qualifier ions generally selected as the two most intense (abundant) ions. Careful consideration was given to ensure the ions chosen were suitably selective (i.e., not produced as a result of a common neutral losses e.g., loss of water (−18 amu) [35]. The quantifier ion was assigned as the most abundant *m*/*z* ion of the two daughters. For the majority, the 2002/657 ion ratio criterion (≤20%) was adhered to, with the exception of a few analytes on a few occasions, where the SANTE criterion (≤30%) was necessary.

#### 2.2.2. Specificity and Linearity

**Figure 2:**
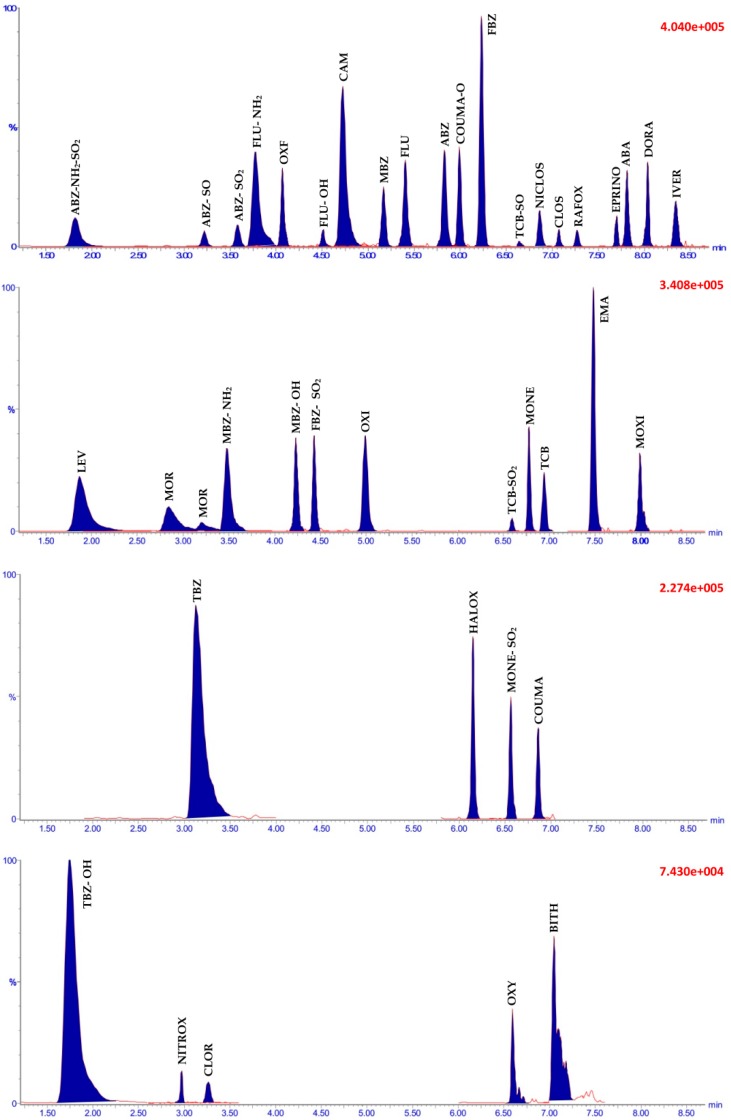
Overlay of LC-MS/MS chromatograms for the 40 anthelmintic residues in a blank water sample fortified at concentrations equivalent to the limit of quantification (LOQ) (see Table 2) for each analyte.

The specificity of the method was investigated through monitoring for interferences in UHPLC-MS/MS traces from analytes or internal standards. Transitions for ABZ-SO_2_ (*m*/*z* 298.1→266.2) and MBZ-OH (*m*/*z* 298.25→266.15) were prone to isobaric interference but were sufficiently separated in the UHPLC-MS/MS traces (3.44 vs. 4.09 min., respectively (Figure 2)). The absence of cross-talk interference was confirmed by injecting analytes and internal standards separately. The selectivity of the method was evaluated by application to 30 different groundwater and surface water samples, which were confirmed to be free of interferences, according to the 2002/657 criterion; however, in some instances, the SANTE criterion (≤30%) was more appropriate. 

Linearity was assessed by visual inspection of these calibration curves (constructed with a linear fit and 1/x^2^ weighting), residual plots and regression coefficient values (R^2^) values. For all analytes, R^2^ values were >0.99, except for TCB-SO and TCB-SO_2_ (0.97 and 0.89) (Table 2). Whelan et al. [36] proposed the use of trifluoroacetic acid (TFA) as a mobile phase additive which allowed better ionisation of these two analytes by promoting the formation of the protonated pseudomolecular ions in ESI positive (+ve) mode. This approach was beyond the scope of this work; therefore, these two analytes are only suitable for screening purposes (non-confirmatory) in this method.

#### 2.2.3. Trueness and Precision

Trueness and precision data under within-lab repeatability (WL_r_) and within-lab reproducibility (WL_R_) conditions are summarised in Table 3. Under WL_r_ conditions the trueness for all analytes was satisfactory and met the set criteria, with overall trueness in the range of 83–113%. WL_r_ precision (RSD_r_) for all analytes across the three validation levels was in the range of 0.8–13.2%, with the exception of NITROX which had an RSD_r_ of 19.5% at the lowest validation concentration, which still met the acceptance criteria. The majority of analytes had RSD_r_ values ≤5%. Under reproducibility conditions (WL_R_), trueness ranged from 88–114%, with all analytes meeting the acceptance criteria. Precision for all analytes under WL_R_ conditions (RSD_wR_) were all under 12.4%, again with the exception of NITROX, which had an RSD of 19.4% at the lowest validated level. Overall this method has been shown to be very accurate and precise for the 38 confirmatory analytes.

#### 2.2.4. Recovery, Limits of Detection (LOD) and Limits of Quantification (LOQ)

The recovery of analytes (Table 3) at the higher concentration (200/400 ng L^−1^) ranged from 71 to 114%, all within the acceptable criteria (70–120%), except for NITROX (56%) and MOXI (59%). The precision for all analytes was <8.7% RSD. At the lower concentration (20/40 ng L^−1^) the overall analyte recoveries ranged from 83–113%, while RSDs ranged from 1.3–11.6%. Notably, the recoveries of NITROX and MOXI were satisfactory at the lower concentration (105 and 95%, respectively). This method performs better (in terms of recovery) when compared to other methods available. Krogh et al. [27] reported a recovery range of 38–67% for ABA, DORA, EMA, EPRINO, IVER and MOXI, using HLB SPE; however, individual recoveries for each analyte could not be clarified throughout the paper. Notably, Krogh et al. used a 4 mL MeOH wash step prior to drying and elution, which may have resulted in removal of analyte at the wash stage. In the method by Zrncic et al. [26], using HLB SPE of water samples at pH 7, recovery ranged between 76.5 and 105.5% for ABZ, FBZ, FLU, MBX, OXI and TCB. Low recoveries of 42.8 and 56.6% were reported for LEV and MOXI respectively. The recovery of LEV reported in this current paper is much higher than that achieved by Zrncic et al., while the recovery of MOXI in this current work performs similarly, or better, depending on analyte concentration (much improved recovery at lower concentration in this work).

The LOQ for the majority of analytes corresponded to the lowest calibrant level of the calibration curve, with an overlaid LC-MS/MS chromatogram for all 40 analytes, fortified in blank water samples at the LOQ, shown in Figure 2. The LOQs ranged from 0.5–10 ng L^−1^, with the exception of EPRINO and CLOR, which had LOQs of 20 and 40 ng L^−1^, respectively. The LOQs for all compounds were lower than 25 ng L^−1^ detection capability required by the EU Drinking Water Directive [19], and given that the method’s LODs are inherently lower than the LOQs, this method more than meets this performance criterion. The exception to this is CLOR, which has an LOQ of 40 ng L^−1^; however, the LOD was determined to be acceptable (10 ng L^−1^). The performance of this method in terms of sensitivity, performs similar to or better (depending on the analyte) than other methods available.

### 2.3. Matrix Effects 

In this study, matrix effects were calculated as follows: ME (%) = (B − A/A × 100), where A is the response of analyte in neat solution, and B is the response in post-extraction spiked samples. Using this approach, negative (−) ME values indicated suppression (decrease in analyte response due matrix components), while positive (+) values indicated enhancement (increase in analyte response). All anthelmintic compounds experienced ion enhancement due to matrix, with the exception of CLOR, CLOS, TBZ-OH and TCB-SO, which all showed ion suppression on average (Table 2). The mean matrix effects (*n* = 30) ranged from -15.1% for CLOR (analyte suppression) up to +93.4% for ABZ-SO (enhancement). The range of ME for each individual analyte across the entire 30 samples is shown in Table 2. The most suppression in any one sample (of total 30) was 74% (ME −74%) for HALOX, while the highest enhancement in any one sample was observed for ABZ-SO (+212%). In order to account for this observed enhancement or suppression due to ME, isotopically labelled internal standards (IS) were employed (IS as specified in Table 2) When the internal standards were incorporated into the method, the overall precision (RSD%) was improved for a number of analytes, particularly ABZ-SO with the RSD reduced from 32% to 7%. In cases where the IS did not drastically improve the precision (e.g., DORA and EMA), the exact deuterated form of the compound was not used as the IS, either due to unavailability or cost, in which case the addition of IS was used only to account for losses of analyte during extraction. Overall the combination of the use of matrix matched calibration curves and internal standards (IS) compensated for any ME effects, thus satisfying validation criteria.

### 2.4. Applicability 

The method presented above has been applied for the determination of the 40 anthelmintic compounds as part of an initial pilot sampling programme, whereby 72 environmental water samples were collected from different locations across Ireland during Autumn 2016 (September–October 2016). Overall, as part of this pilot study, 52 groundwaters (from boreholes, wells and springs) and 20 surface waters (from streams, rivers and lakes) were collected from 43 different sampling locations and analysed for the 40 anthelmintic compounds. Anthelmintic compounds were detected in 8 out the 72 samples (11%) with concentrations of the order of 1.0 ng L^−1^ to 30 ng L^−1^. Of the eight samples with detections, four were groundwater samples which contained up to three different anthelmintic compounds (detection in 7.7% of groundwater samples analysed), while the other four were surface waters with up to five different anthelmintics present (detections in 20% of surface waters analysed). The method has also been applied in more comprehensive spatial and temporal studies, which are currently in preparation.

## 3. Materials and Methods 

### 3.1. Chemicals, Standards and Consumables

Ultrapure water (UPW) (18.2 MΩcm) was generated in house using a Millipore water purification system (Cork, Ireland). Romil “SpS” (super purity solvent) grade methanol (MeOH) 215, acetonitrile (MeCN) 200 far UV and propan-2-ol (IPA) were sourced from Romil Ltd. (Cambridge, UK). Acetone puriss was purchased from Honeywell Research Chemicals (Honeywell Riedel-de Haen; Seelze, Germany). Dimethyl sulfoxide (DMSO), 99.5% d-MeOH, ammonium formate puriss p.a. (puriss pro analysis), formic acid (HCOOH) 98-100%, methyl tert-Butyl ether (MTBE) (Fluka for GC) and sodium meta-bisulphite (>97%) were sourced from Sigma-Aldrich (Dublin, Ireland). Glacial Acetic acid (CH_3_COOH) (100%) and ammonia solution (25% *w*/*v*) were obtained from Merck (Darmstadt, Germany). Concentrated hydrochloric acid (HCl) (36%) was sourced from BDH Chemicals Ltd. (Poole, UK). 

Neat analytical standards of abamectin (ABA), albendazole (ABZ), bithionol (BITH), clorsulon (CLOR), closantel (CLOS), coumaphos (COUMA), doramectin (DORA), Emamectin benzoate (EMA), eprinomectin (EPRINO), fenbendazole (FBZ), haloxon (HALOX), ivermectin (IVER), levamisole hydrochloride (LEV), morantel-tartrate-hydrate (MOR), moxidectin (MOXI) niclosamide (NICLOS), nitroxynil (NITROX), oxfendazole (OXF), oxyclozanide (OXY), rafoxanide (RAFOX), thiabendazole (TBZ), triclabendazole (TCB) and salicylanilide (SAL) were purchased from Sigma-Aldrich Ireland (Dublin, Ireland). Albendazole-sulphoxide (ABZ-SO), albendazole-sulphone (ABZ-SO_2_), albendazole-amino-sulphone hydrochloride (ABZ-NH_2_-SO_2_), cambendazole (CAM), fenbendazole-sulphone (FBZ-SO_2_), 5-hydroxy-thiabendazole (5-OH-TBZ), triclabendazole sulphoxide (TCB-SO), triclabendazole sulphone (TCB-SO_2_) and amino-triclabendazole (TCB-NH_2_) were purchased from Witega (Berlin, Germany). Coumaphos-oxon (COUM-O) was purchased from Greyhound Chromatography and Allied Chemicals, (Merseyside, UK). Flubendazole (FLU), amino-flubendazole (FLU-NH_2_), hydroxy-flubendazole (FLU-OH), mebendazole (MBZ), amino-mebendazole (MBZ-NH_2_) and hydroxy-mebendazole (MBZ-OH) were obtained from Janssen Animal Health (Beerse, Belgium). Oxibendazole (OXI) was purchased from QMX Laboratories (Essex, UK), while selamectin (SEL) was acquired from Pfizer (Kent, UK). Monepantel (MONE) and monepantel-sulphone (MONE-SO_2_) were purchased from Novartis Pharmaceuticals (Dublin, Ireland). All deuterated or isotopically labelled internal standards (specified for each compound in Table 2) were purchased from Witega (Berlin, Germany), except for flubendazole-d_3_ (FLU-d_3_) which was purchased from Sigma-Aldrich Ireland (Dublin, Ireland).

Duran style (GL45) glass amber bottles (1000 mL) were purchased from Scientific and Chemical Supplies Ltd. (Cork, Ireland). Analytical grade glass wool (silanised and unsilanised) were purchased from Sigma-Aldrich (Dublin, Ireland). Polypropylene tubes (15 mL, conical) were obtained from Sarstedt Ltd. (Wexford, Ireland). Isolute 150 mL frittless SPE reservoirs were purchased from Biotage (Uppsala, Sweden). Reservoirs were connected to the SPE cartridge using adapter caps for 1–6 mL cartridges, provided by Agilent Technologies Ltd. (Cork, Ireland). Captiva Econo PTFE 0.2 µm filters were also purchased from Agilent Technologies Ltd., as were the glass inserts (400 µL) used in the Waters HPLC vials (Waters; Dublin, Ireland) The different SPE sorbents evaluated as part of method development included: Bond Elut ENV (200 mg, 6 mL) and Bond Elut PLEXA (200 mg, 6 mL) from Agilent technologies Ltd. (Cork, Ireland), Oasis HLB (200 mg, 6 mL) from Waters (Dublin, Ireland) and UCT Enviro Clean HL DVB (200 mg, 6 mL) and UCT Enviro Clean HL DVB (500 mg, 6 mL) from United Chemical Technologies Ireland Ltd.

### 3.2. Preparation of Standard Solutions 

Individual primary stock solutions were prepared from certified standard material at a concentration of 4 mg mL^−1^ in MeCN for EPRINO, in MeOH for BITH, CLOR, CLOS, MOR, NITROX and OXY and in DMSO for ABZ, ABZ-SO, ABZ-SO_2_, ABZ-NH_2_-SO_2_, FBZ, FBZ-SO_2_, MONE, MONE-SO_2_ and OXF. Stock solutions at a concentration of 2 mg mL^−1^ were prepared in MeCN for ABA, DORA, EMA, IVER, MOXI and SEL, in MeOH for CAM, COUMA, COUMA-O, HALOX, LEV, NITROX, RAFOX, TBZ, TCB, TCB-SO, TCB-SO_2_ and TCB-NH_2_ and in DMSO for FLU, FLU-OH, FLU-NH_2_, MBZ, MNZ-OH, MBZ-NH_2_, OXI and TBZ-OH. Ten mixed intermediate solutions were prepared in MeOH as follows: WS-A containing 50 µg mL^−1^ CLOR, WS-B containing 50 µg mL^−1^ of ABA, EPRINO, IVER and MOXI, WS-C containing 25 µg mL^−1^ DORA and NITROX, WS-D containing 25 µg mL^−1^ of EMA, MONE-SO_2_, NICLOS and TBZ-OH, WS-E containing 25 µg mL^−1^ of CLOS, FLU-OH, FLU-NH_2_ and RAFOX, WS-F containing 25 µg mL^−1^ of ABZ, ABZ-SO, ABZ-SO_2_, ABZ-NH2-SO_2_, CAM, COUMA-O, FBZ, FBZ-SO_2_, FLU, LEV, MBZ, MBZ-OH, MBZ-NH_2_, MOR, OXF, OXI, TBZ and TCB, WS-G containing 10µg mL^−1^ of TCB-SO, WS-H containing 10 µg mL^−1^ of TCB-SO2, WS-I containing 25 µg mL^−1^ of BITH, HALOX and OXY, and WS-J containing 25 µg mL^−1^ of MONE and COUMA.

A set of seven mixed working calibration solutions (Calibrants 1–7) with concentration ranges of 100–2500 ng mL^−1^, 50–2500 ng mL^−1^, 25–1250 ng mL^−1^, 2.5–500 ng mL^−1^, 5–1250 ng mL^−1^, 2.5–1250 ng mL^−1^, 12.5–1250 ng mL^−1^ and 12.5–500 ng mL^−1^ were prepared in MeOH by dilution of the respective intermediate mixed working solution; WS-A to WS-F and WS-I to WS-J. For TCB-SO and TCB-SO_2_, an intermediate calibration solution (INT-A) at a concentration of 20 ng mL^−1^ was prepared by dilution of WS-G and WS-H. Primary stock of all deuterated and labelled internal standards, in addition to SAL, were prepared at a concentration of 1 mg mL^−1^. These single stocks were subsequently used to prepare an intermediate IS solution in deuterated MeOH containing 200 µg mL^−1^ SEL and TCB-NH_2_, 40 µg mL^−1^ LEVA-d_5_, TBZ-^13^C_6_ and SAL, and 20 µg mL^−1^ of all other deuterated/labelled internal standards. This intermediate IS solution was diluted 1 in 10 to give a 20/4/2 µg mL^−1^ working IS solution. All working solutions were stored at −18 °C or, below in glass amber vials, with equilibration to room temperature before use. 

### 3.3. Sample Collection, Control Samples and Quality Control (QC) 

Water samples were collected (in 2.5 L amber bottles) by one of three techniques depending on the source: (a) traditional grab sampling direct into the sampling container; (b) grab sampling via a bailer device, or (c) by pump (peristaltic or submersible). The sampling container was rinsed three times with the source water prior to collection. Samples were transported to the laboratory under chilled conditions, in individual, sealed, polypropylene bags and stored at 4 °C until analysis (within 7–10 days after collection, as determined by matrix stability studies).

Samples found to be free of analyte, or to contain analyte levels of <30% of the lowest calibration point (in accordance with SANTE [38]), were deemed to be suitable as negative control samples for method development, matrix matched calibration and validation experiments. Negative control samples were also used to produce QC Trip (Field) blanks. A QC trip blank (500 mL negative control aliquot) was transported to and from field sites while sampling. In the field, at each sampling location, the trip blank was exposed (open capped) in the vicinity of the sampling point, for the duration of sampling, and accompanied samples back to the laboratory in the same cooler container and under the same conditions. This trip blank was subsequently analysed along with samples, to demonstrate there was no contamination of samples during collection and transport. Fortified QC samples were not used during this study as some sampling was carried out by external organisations, and fortified samples were not feasible in such cases.

For internal (within batch) QC, a system suitability check to monitor analyte response and retention was injected prior to each instrumental run, to ensure the instrument was performing as expected. Negative control samples (*n* = 2) were included to confirm no cross contamination during the extraction process. Post-extraction spiked recovery samples were included to ensure the performance of the method for each analyte. Solvent blank injections were incorporated following calibration samples, prior to injection of unknown samples, to demonstrate no carryover of analytes. Retention checks involved re-injection of a matrix calibrant several times throughout the analytical run to check for accuracy. A minimum of four retention check injections were used to ensure no drift in retention during the analytical run, and to ensure no variation in detector response.

### 3.4. Matrix Matched Calibration 

Matrix matched calibration curves were prepared by fortification of negative control samples as described in Table 4. An additional lower and upper calibration point was produced for some analytes. A minimum of seven points was used to construct a calibration curve, with the individual calibration range for each analyte as shown in Table 2. For TCB-SO and TCB-SO_2_, a calibration curve was prepared by spiking of respective calibration samples above with 50, 100, 150, 250, 350, 450, 500, 550 and 625 µL of INT-A to give the concentrations described in Table 4 (analyte group G and H). All calibrants, quality control samples and samples were fortified with internal standard (25 µL) corresponding to a sample concentration 1000 ng L^−1^ SEL and TCB-NH_2_, 200 ng L^−1^ LEVA-d_5_, TBZ- ^13^C_6_ and SAL, and 100 ng L^−1^ of all other deuterated/labelled internal standards. 

### 3.5. UHPLC-MS/MS Determination 

All analytes were chromatographically separated using an in-house method as previously described by Whelan et al., 2010 [30]. Here analytes were separated on a stainless steel HSS T3 (100 mm × 2.1 mm, 1.8 μm particle size) column on a Waters Acquity UHPLC system, with a binary gradient. Anthelmintic residues were detected by a Waters Quattro Premier XE triple quad mass spectrometer (Milford MA, USA) with an electrospray ionisation (ESI) interface, coupled to the LC. All analysis was performed using rapid polar switching using a modified version of the acquisition described by [30]. Dwell times, collision energies (CE) and collision voltages (CV) were further optimised from the original method, with the modified conditions shown in Supplementary Information Table S2.

### 3.6. Sample Preparation-Solid Phase Extraction

#### 3.6.1. Development and Optimisation 

**Figure 3:**
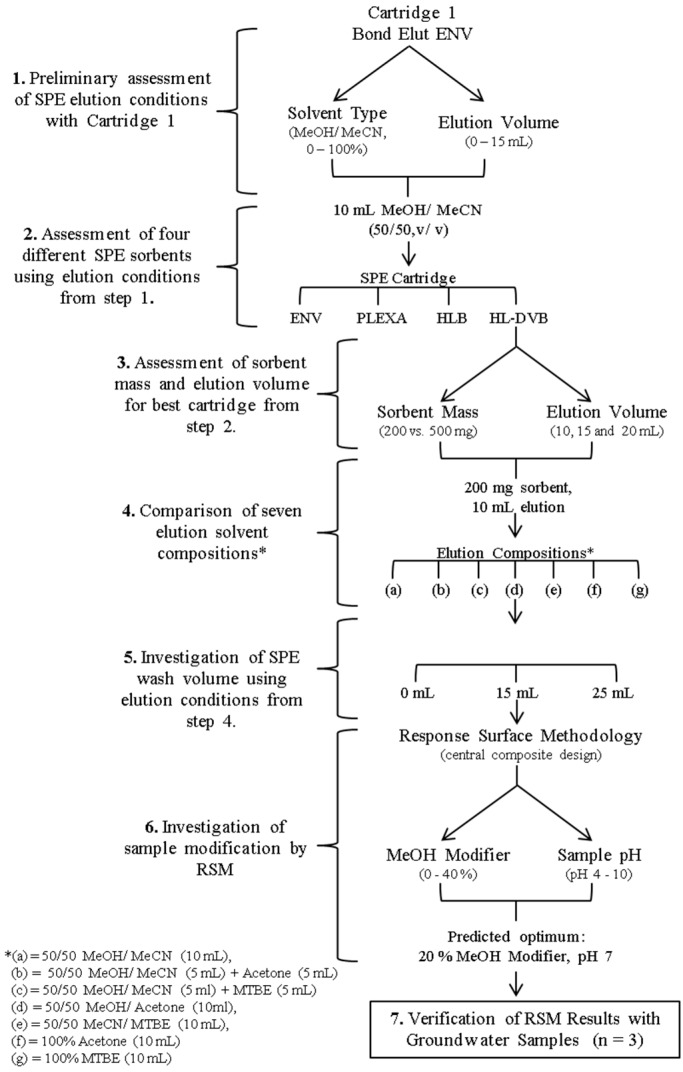
Summary of experimental conditions carried out for the response surface model.

The main experiments carried out for SPE optimisation are summarised in Figure 3. All experiments were performed by fortification (*n* = 3) of negative control water samples, giving a concentration of 200 ng L^−1^ for analytes except BITH, CLOR, MOR and OXY, which were fortified at 400 ng L^−1^. All SPE cartridges were conditioned and equilibrated according to the final procedure as described below. For experiments 1 to 4, the SPE cartridges were washed with ultrapure water, with experiments proceeding experiment 5 incorporating the selected optimum wash solution. In experiment 6, a simple central composite design response surface methodology (RSM) experiment was employed to optimise sample pretreatment steps. The experimental design was carried out using MiniTab^®^ 17 Statistical Software version 17.1.0 (MiniTab Inc., PA, USA) This experiment investigated two independent factors: (a) MeOH modifier added to samples (0–40%) and (b) sample extraction pH (pH 4–10). A quadratic model was selected to generate 13 experimental combinations (Supplementary Table S3), including five central combinations to assess error within the model. In the first stage, data was acquired and evaluated for all 40 analytes, with 17 analytes further evaluated at Stage 2 using an RSM optimiser graph, to optimise these two sample modification factors. These 17 analytes were selected to include different anthelmintic compounds representative of the different structural classes, in addition to the analytes which demonstrated poor recoveries in previous experiments (e.g., CLOS and RAFOX). Predicted results were verified by the optimiser graphs for the remaining 23 of the total 40 analytes (Supplementary Figure S3b).

#### 3.6.2. Final Method 

Water samples (500 ± 0.1 g corresponding to 500 ± 0.1 mL), preshaken, were weighed directly into glass amber bottles (1000 mL) and equilibrated to room temperature. Matrix calibrants and samples were fortified with the working calibrant and IS solutions as described in Section 3.4. These were then shaken (60 s), modified with MeOH (100 mL), and shaken again (1 min). Samples were subsequently adjusted to pH 7 ± 0.05 with HCl (0.1 M) or NH_4_OH (0.1 M). The sample-modifier mixtures (600 mL) were purified on UCT Enviro Clean HL DVB (200 mg, 6 mL) SPE cartridges packed with glass wool (2.5 ± 0.2 g). Prior to loading, SPE cartridges were conditioned with MeOH: Acetone (50: 50, *v*/*v*) (5 mL) and MeOH (5 mL) and equilibrated with ultrapure water, pH 7 (5 mL). Samples were loaded under vacuum through large volume reservoirs (150 mL) on top of the SPE cartridge, at a rate of 6 mL min^−1^. Once loaded, samples bottles were rinsed with H_2_O: MeOH (90:10, *v*/*v*) (10 mL) and added to the SPE. The SPE cartridge was then washed with a further 5 mL aliquot of H_2_O: MeOH (90:10, *v*/*v*). Cartridges were dried under vacuum (30 min) and eluted with MeOH: Acetone (50:50, *v*/*v*) (10 mL) into 15 mL polypropylene tubes. DMSO (500 µL) was added to each sample as a keeper solvent and vortexed (30 s). Samples were evaporated under nitrogen using a TurboVap LV (50 °C, 15–20 psi, 60–90 min) until 500 µL DMSO remained. Extracts were sonicated (2 min.) and vortexed (30 s) prior to filtration through 0.22 µm syringe filters into glass HPLC vials for instrumental determination.

### 3.7. Method Validation Procedure

There are currently no legislative guidelines available for validation of veterinary residues in water matrix; therefore, a method validation approach was implemented based on a combination of criteria set out in SANTE/11813/2017 guidelines [38] relating to pesticides in food and European Legislation 2002/657/EC [37], pertaining to veterinary residues in food. As part of this validation the following performance parameters were examined: identification, selectivity, sensitivity/linearity, trueness, within-laboratory repeatability (WL_r_ or RSD_r_) and within-laboratory reproducibility (WL_R_ or RSD_wR_). Further to this, method recovery, limits of detection (LOD) and limits of quantification (LOQ) were also assessed as part of method validation. Validation was performed at concentration levels equivalent to a low, medium and high concentration across the calibration curve, to be consistent with the method sensitivities for the different analytes (described in Table 2). 

The selectivity of the method was assessed by individually injecting standards and internal standards to check for isobaric interferences by monitoring all transitions. In addition, blank groundwater samples (*n* = 30, all from different sources) were analysed along with reagent blanks (both spiked with IS and non-spiked with IS) to determine any matrix interferences co-eluting with analytes. To assess linearity, matrix matched calibration curves, with at least seven points, were prepared by fortification of negative controls over a range of concentrations as described above (Table 4). 

Trueness and Precision were both assessed in terms of within lab repeatability conditions (WL_r_) and within lab reproducibility (WL_R_) conditions, using fortified negative control samples, given that no certified reference material is available for these analytes in water. The WL_r_ study involved a negative control sample fortified at each of three validation levels in replicates of *n* = 6. For WL_R_ a similar experiment was carried out at the same three concentration levels, with a total of *n* = 18 replicates analysed over 5 different days (3 days with *n* = 4 replicates and 2 day with *n* = 3 replicates). In this case, negative control samples from different sources were used on each of the different days, with different negative controls also used for each of the three validation levels. 

The dependence of recovery on analyte concentration was assessed whereby blank water samples were fortified pre- and post-extraction (*n* = 3) at two different concentrations; 20 ng L^−1^ and 200 ng L^−1^ for all analytes, except BITH, CLOR, MOR and OXY, which were at concentrations of 40 and 400 ng L^−1^. Recovery was determined by comparison of analyte response in the pre-extraction spiked samples (spiked at the beginning, immediately prior to extraction) to that in the samples spiked post-extraction (spiked at the end, immediately prior to instrumental determination). Use of such approach allowed the effects of matrix on analyte response to be considered in calculating the recovery. 

LODs and LOQs were determined by fortification of blank samples at concentrations equivalent to the lowest calibrant level. The chromatographs of each analyte, on five different occasions, were visually inspected and the LOD and LOQ were given as the estimated analyte concentration that achieved a signal to noise (S/N) of 3 and 10, respectively, with consideration given to both quantifier and qualifier ions. The LOQ was assessed as the lowest spiking level which satisfied the method performance criteria set out by SANTE for trueness and precision, in combination with the minimum S/N.

### 3.8. Matrix Effects

Matrix effects (ME) were assessed using the post-extraction spiking method adapted from Matuszewski et al. [39] with matrix effects calculated as follows: ME (%) = (B − A/A × 100), where (A) is the response of analyte in neat solvent, and (B) is the response of analyte in matrix extract, spiked post-extraction. Negative control samples (*n* = 30), from different groundwater and surface water sources in Ireland (spring, boreholes, streams and lakes), were extracted and spiked post-extraction corresponding to a concentration of 100 ng L^−1^ for all analytes, except BITH, CLOR, MOR and OXY at 200 ng L^−1^. These post-extraction spiked samples were compared to solvent standards to quantify the ion enhancement or suppression due to matrix. Using this approach, negative (−) ME values indicated suppression (decrease in analyte response due to endogenous and/or exogenous matrix components), while positive (+) values indicated enhancement (increase in analyte response due to matrix components).

## 4. Conclusions

A comprehensive and sensitive analytical method, based on SPE followed by LC–MS/MS detection, has been developed for the quantitative confirmatory analysis of 38 anthelmintic compounds in raw, unfiltered, environmental water samples and screening analysis for a further two anthelmintic residues. The method has been extensively validated over a broad range of concentration levels, in-line with expected concentration in the environment, based on review of currently available literature. This method is advantageous compared to existing analytical methods for environmental samples because it allows for analysis of a wider range of anthelmintic residues (40), from different structural classes. Of these 40 compounds, 13 of them are metabolites/transformation products, for which currently available methods are lacking. This provides a more comprehensive application to improve the understanding of the environmental occurrence of anthelmintics. The method development work carried out showed the impact of sample modification prior to extraction, which in this case aided desorption of some analytes from the sample container. The matrix effect study demonstrated the importance of assessing ion enhancement/suppression due to matrix, as part of method development and validation stages. The results of this study highlighted the significance of incorporating deuterated internal standards into the analytical methodology, which was shown to improve the overall accuracy and precision for the majority of analytes. This work incorporates deuterated or surrogate internal standards for all 40 compounds. The overall method presented was validated according to appropriate guidelines and deemed to be fit for the purpose intended. 

## Figures and Tables

**Table 1:** Validation criteria adhered to, with corresponding legislative guideline.

Parameter	Performance Criteria	Guideline ^a^
**Identification**		
Points	Minimum 3	2002/657
Relative retention (RRT)	≤2.5%	2002/657
Ion ratio tolerance (ΔR)	20–50% 30%	2002/657 SANTE
**Selectivity**	Interferences: ≤10% lowest calibrant Interferences: ≤30% lowest calibrant	2002/657 SANTE
**Linearity**	Regression coefficient R^2^ ≥ 0.98 Residuals ± 20%	2002/657 SANTE
**Trueness (WL_R_ and WL_r_)**	70–120%	SANTE
**Precision (RSD_wR_ and RSD_r_)**	≤20%	SANTE
**Recovery**	70–120%	SANTE

^a^ 2002/657 = European Commission Decision 2002/657/EC [37], SANTE = SANTE/11813/2017 [38].

**Table 2:** Calibration range, mean linearity (of *n* = 5 runs), and results of matrix effects (ME) (*n* = 30) for each of the 40 anthelmintic compounds.

Analyte	Abbreviation	P/TP	Labelled IS Used	Calibration Range (ng L^−1^)	Linearity R^2^	Mean ME (%) (*n* = 30)	ME RANGE (%)		RSD No IS (*n* = 30) (%)	RSD with IS (*n* = 30) (%)
							Min	Max		
**Benzimidazoles**										
Albendazole	ABZ	P	ABZ-d_3_	1–1000	0.997	27.1	8.2	47.3	9.3	3.0
Albendazole sulphoxide	ABZ-SO	TP	ABZ-SO-d_3_	1–1000	0.994	93.4	13.8	212	31.6	7.1
Albendazole sulphone	ABZ-SO_2_	TP	ABZ-SO_2_-d_3_	1–1000	0.996	60.8	29	120	18.2	6.5
Albendazole-amino-sulphone	ABZ-NH_2_-SO_2_	TP	ABZ-NH_2_-SO_2_-d_3_	0.5–1000	0.998	16.9	−1.4	28.0	6.9	4.0
Cambendazole	CAM	P	FBZ-d_3_	0.5–1000	0.997	9.7	−5.1	24.2	6.6	7.2
Fenbendazole	FBZ	P	FBZ-d_3_	0.5–1000	0.995	23.1	1.0	44.9	9.3	2.3
Oxfendazole	OXF	TP	FBZ-SO-d_3_	1–1000	0.993	42.0	11.6	106.2	18.5	6.4
Fenbendazole sulphone	FBZ-SO_2_	TP	FBZ-SO_2_-d_3_	1–1000	0.998	47.5	8.1	165.7	25.5	3.3
Flubendazole	FLU	P	FLU-d_3_	1–1000	0.996	33.3	7.4	108.2	14.1	3.7
Amino-flubendazole	FLU-NH_2_	TP	TCB-NH_2_ (pos)	1–1000	0.995	11.5	−3.7	29.8	8.0	8.8
Hydroxy-flubendazole	FLU-OH	TP	MBZ-OH-d_3_	1–1000	0.997	3.7	−12.9	27.4	12.1	7.6
Mebendazole	MBZ	P	MBZ-d_3_	1–1000	0.994	45.0	11.4	104.2	18.1	3.6
Amino-mebendazole	MBZ-NH_2_	TP	TCB-NH_2_ (pos)	1–1000	0.995	15.1	0	36.3	7.2	8.5
Hydroxy-mebendazole	MBZ-OH	TP	MBZ-OH-d_3_	1–1000	0.998	27.4	3.8	64.2	13.1	4.6
Oxibendazole	OXI	P	OXI-d_7_	0.5–1000	0.994	9.3	−2.5	21.6	5.8	4.5
Triclabendazole	TCB	P	TCB-d_3_	0.5–1000	0.997	3.6	−14.2	27.4	8.0	3.3
Triclabendazole-sulphoxide	TCB-SO	TP	TCB-NH_2_(neg)	4–20	0.967	−3.0	−45.0	47.8	25.2	24.7
Triclabendazole-sulphone	TCB-SO_2_	TP	TCB-NH_2_(neg)	4–20	0.891	5.2	−25.4	57.8	18.2	19.8
Thiabendazole	TBZ	P	TBZ-^13^C_6_	0.5–1000	0.999	9.1	−6.8	26.6	6.7	2.7
5-Hydroxy-Thiabendazole	TBZ-OH	TP	ABZ-NH_2_-SO_2_-d_3_	0.5–200	0.991	−6.4	−23.8	12.7	9.6	7.2
**Macrocyclic lactones (Avermectins & Milbemycins)**										
Abamectin	ABA	P	SEL	10–2000	0.996	20.4	−4.1	45.7	9.7	7.5
Doramectin	DORA	P	SEL	10–1000	0.993	77.8	13.8	130.9	16.0	15.2
Emamectin	EMA	P	SEL	0.5–200	0.996	24.8	3.4	37.8	7.7	8.2
Eprinomectin	EPRINO	P	SEL	20–2000	0.997	6.8	−17.9	25.7	9.9	8.3
Ivermectin	IVER	P	SEL	10–2000	0.996	5.2	−22.5	27.2	9.6	7.9
Moxidectin	MOXI	P	SEL	10–2000	0.996	34.9	−9.1	76.3	16.3	13.7
**Salicylanilides and substituted phenols**										
Bithionol	BITH	P	RAFOX-^13^C_6_	5–1000	0.995	32.0	−1.4	50	10.6	5.4
Closantel	CLOS	P	CLOS-^13^C_6_	2–1000	0.997	−3.9	−12.1	5.2	5.4	2.8
Niclosamide	NICLOS	P	SAL	1–200	0.991	13.0	−5	33.3	8.3	5.1
Nitroxynil	NITROX	P	NITROX-^13^C_6_	10–1000	0.993	28.6	−5.7	73.2	14.9	14.2
Oxyclozanide	OXY	P	OXY-^13^C_6_	5–1000	0.996	42.8	18.2	70.9	9.5	10.4
Rafoxanide	RAFOX	P	RAFOX-^13^C_6_	2–1000	0.994	23.0	2	41.2	10.5	3.4
**Tetrahydropyrimidines**										
Morantel	MOR	P	TBZ-^13^C_6_	1–1000	0.997	13.3	−2.5	34.1	7.4	1.6
**Imidazothiazoles**										
Levamisole	LEV	P	LEVA-d_5_	0.5–1000	0.999	12.4	−2.5	33.7	7.4	2.0
**Organophosphates**										
Coumaphos	COUMA	P	ABZ-d_3_	5–200	0.986	47.0	10.1	87.7	12.9	8.4
Coumaphos-Oxon	COUMA-O	P	FBZ-d_3_	1–1000	0.992	16.2	3.9	31.9	6.0	7.6
Haloxon	HALOX	P	ABZ-d_3_	5–500	0.989	25.5	−73.8	55	12.0	7.2
**Amino-acetonitrile derivatives**										
Monepantel	MONE	P	CLOS-^13^C_6_	5–400	0.991	16.7	−6.1	31.8	7.2	8.5
Monepantel-sulphone	MONE-SO_2_	TP	CLOS-^13^C_6_	1–400	0.993	14.0	−4.4	28.8	7.0	7.4
**Miscellaneous**										
Clorsulon	CLOR	P	SAL	40–2000	0.991	−15.1	−48.8	9.2	18.7	15.6

P = Parent compound, TP = Transformation product, IS = Internal standard, R^2^ = regression coefficient, ME = Matrix effects where positive values indicate ion enhancement, while negative values indicate ion suppression. Matrix effect study was carried out at a concentration of 100 ng L^−1^ for all analytes except CLOR, BITH and MOR, which were at 200 ng L^−1^ RSD = relative standard deviation.

**Table 3:** Validation trueness and precision (RSD) under repeatability conditions (WLr) (*n* = 6) and reproducibility conditions (WLR) (*n* = 18) at three concentration levels for 40 anthelmintics with respective method recovery, LOD and LOQ values (ng L^−1^).

Analyte	Validated Levels L1, L2, L3 (ng L^−1^)	WL_r_ Trueness (RSD_r_) (%) ^a^			WL_R_ Trueness (RSD_WR_) (%) ^b^			LOD ^c^ (ng L^−1^)	LOQ ^d^ (ng L^−1^)	Recovery % (RSD%, *n* = 3) at	
		L1	L2	L3	L1	L2	L3			20/40 ng L^−1^	200/400 ng L^−1^
**Benzimidazoles**											
ABZ	5, 50, 200	100 (5.6)	100 (3.0)	97 (1.5)	102 (3.6)	100 (3.2)	98 (2.8)	0.125	1.0	94 (4.7)	94 (0.5)
ABZ-SO	5, 50, 200	113 (10.8)	97 (7.3)	101 (4.7)	107 (13.5)	99 (9.9)	99 (5.2)	0.2	1.0	95 (1.3)	114 (5.5)
ABZ-SO2	5, 50, 200	95 (7.3)	96 (4.5)	99 (2.5)	105 (6.4)	99 (3.2)	99 (3.2)	0.165	1.0	92 (2.1)	105 (5.6)
ABZ-NH2-SO2	5, 50, 200	103 (3.1)	101 (1.4)	101 (1.1)	101 (3.7)	99 (2.3)	100 (3.9)	0.165	0.5	93 (4.0)	91 (7.6)
CAM	5, 50, 200	103 (4.0)	96 (1.4)	97 (1.1)	102 (4.3)	101 (3.9)	100 (3.1)	0.165	0.5	94 (3.2)	92 (6.0)
FBZ	5, 50, 200	103 (4.5)	97 (2.0)	100 (1.3)	105 (6.7)	100 (3.8)	99 (2.3)	0.1	0.5	89 (4.6)	109 (1.9)
OXF	5, 50, 200	87 (11.4)	100 (5.4)	101 (3.3)	101 (15.1)	98 (6.3)	98 (6.4)	0.25	1.0	94 (6.5)	103 (4.8)
FBZ-SO2	5, 50, 200	99 (2.7)	96 (1.6)	97 (0.8)	101 (5.1)	99 (3.0)	99 (1.7)	0.20	1.0	97 (3.2)	102 (5.5)
FLU	5, 50, 200	107 (7.2)	95 (5.5)	95 (2.1)	102 (7.1)	97 (4.3)	100 (3.3)	0.1	1.0	97 (4.9)	97 (2.5)
FLU-NH2	5, 50, 200	107 (3.6)	104 (3.4)	97 (2.4)	105 (4.8)	103 (2.9)	98 (3.4)	0.05	1.0	94 (5.1)	102 (1.8)
FLU-OH	5, 50, 200	97 (6.8)	109 (4.4)	103 (2.3)	99 (5.6)	102 (4.3)	101 (3.1)	0.3	1.0	95 (4.3)	99 (3.7)
MBZ	5, 50, 200	105 (5.3)	99 (3.6)	97 (2.0)	102 (6.1)	97 (3.9)	98 (2.6)	0.125	1.0	97 (4.0)	102 (0.9)
MBZ-NH2	5, 50, 200	104 (3.4)	104 (3.1)	96 (3.8)	105 (4.8)	104 (3.5)	100 (4.1)	0.3	1.0	92 (2.0)	101 (2.4)
MBZ-OH	5, 50, 200	102 (2.6)	107 (1.0)	100 (1.0)	103 (4.3)	101 (4.2)	99 (2.5)	0.2	1.0	96 (3.6)	104 (5.2)
OXI	5, 50, 200	102 (2.7)	99 (2.7)	97 (1.0)	106 (5.2)	101 (3.3)	98 (3.2)	0.125	0.5	103 (3.3)	98 (2.4)
TCB	5, 50, 200	96 (6.9)	105 (4.5)	102 (3.5)	100 (7.6)	102 (3.5)	100 (3.4)	0.125	0.5	91 (2.0)	100 (4.0)
TCB-SO	6, 14, 20	-	-	-	-	-	-	1.0	4.0	80 (4.8)	92 (6.6)
TCB-SO2	6, 14, 20	-	-	-	-	-	-	1.0	4.0	97 (7.5)	103 (4.8)
TBZ	5, 50, 200	102 (3.8)	99 (1.0)	98 (0.6)	103 (3.2)	99 (2.4)	100 (2.0)	0.1	0.5	99 (3.1)	98 (3.2)
TBZ-OH	5, 50, 150	110 (1.5)	101 (1.2)	93 (0.7)	109 (3.3)	100 (2.1)	92 (4.1)	0.1	0.5	104 (2.0)	80 (5.1)
**Macrocyclic lactones (Avermectins & Milbemycins)**											
ABA	40,150,500	104 (5.4)	99 (5.0)	98 (7.3)	98 (8.5)	100 (5.6)	99 (3.2)	1.0	10.0	110 (9.0)	90 (6.0)
DORA	20, 80, 200	103 (4.7)	97 (5.3)	103 (4.3)	98 (7.9)	97 (7.3)	99 (4.5)	0.5	10.0	105 (6.8)	87 (1.5)
EMA	5, 50, 150	107 (4.5)	96 (9.6)	104 (8.7)	108 (5.6)	104 (6.5)	102 (5.5)	0.05	0.5	102 (5.0)	87 (4.5)
EPRINO	40, 150, 500	96 (3.4)	99 (4.9)	104 (2.6)	100 (8.9)	101 (3.1)	102 (2.4)	5	20.0	109 (0.8)	91 (5.6)
IVER	40, 150, 500	104 (4.1)	100 (2.7)	107 (5.4)	98 (7.5)	100 (2.9)	103 (4.6)	2.5	10.0	113 (10.9)	72 (8.7)
MOXI	40, 150, 500	96 (6.4)	92 (8.7)	91 (6.5)	101 (7.8)	100 (8.0)	98 (6.5)	2.0	10.0	95 (10.8)	59 (5.0)
**Salicylanilides and substituted phenols**											
BITH	20, 80, 200	112 (5.6)	112 (4.7)	104 (2.7)	114 (7.2)	106 (4.8)	101 (3.8)	1.0	5.0	98 (10.8)	84 (3.7)
CLOS	5, 50, 200	105 (4.8)	104 (2.0)	101 (1.0)	105 (7.1)	101 (3.7)	99 (3.2)	0.5	2.0	103 (3.6)	76 (3.5)
NICLOS	5, 50, 150	107 (10.3)	106 (3.7)	96 (2.0)	114 (9.5)	105 (7.2)	96 (6.9)	0.125	1.0	94 (7.0)	100 (5.4)
NITROX	20, 80, 200	107 (19.5)	107 (13.2)	91 (4.6)	96 (19.4)	104 (12.4)	96 (8.7)	2.5	10.0	105 (4.6)	56 (4.7)
OXY	20, 80, 200	113 (6.7)	108 (7.4)	101 (2.4)	109 (9.6)	103 (8.6)	101 (4.1)	1.5	5.0	93 (7.7)	104 (5.6)
RAFOX	5, 50, 200	105 (8.7)	101 (3.0)	99 (1.8)	102 (10.3)	102 (4.3)	101 (2.5)	0.3	2.0	97 (5.8)	86 (4.8)
**Tetrahydropyrimidines**											
MOR	5, 50, 200	101 (1.8)	98 (1.4)	95 (1.8)	100 (2.3)	97 (1.9)	98 (2.8)	0.3	1.0	100 (4.0)	100 (2.5)
**Imidazothiazoles**											
LEV	5, 50, 200	102 (1.5)	100 (1.4)	100 (0.7)	102 (2.1)	100 (1.1)	101 (1.7)	0.125	0.5	89 (5.7)	96 (1.9)
**Organophosphates**											
COUMA	10, 50, 150	83 (9.3)	93 (2,9)	104 (3.8)	88 (8.3)	95 (5.8)	106 (4.7)	1.0	5.0	84 (6.0)	99 (3.6)
COUMA-O	5, 50, 200	95 (3.7)	89 (3.9)	98 (1.6)	96 (6.6)	92 (3.4)	99 (3.2)	0.25	1.0	93 (5.6)	102 (2.5)
HALOX	20, 80, 200	94 (11.7)	94 (3.6)	100 (2.0)	90 (11.8)	94 (5.3)	102 (3.1)	1.0	5.0	83 (0.8)	99 (0.6)
**Amino-acetonitrile derivatives**											
MONE	10, 50, 150	103 (5.1)	96 (4,3)	93 (3.2)	104 (12.1)	97 (6.0)	94 (5.2)	0.5	5.0	90 (6.9)	96 (3.0)
MONE-SO2	5, 50, 150	94 (8.1)	91 (6.2)	93 (3.2)	98 (8.9)	94 (4.6)	98 (5.3)	0.2	1.0	92 (2.6)	102 (1.7)
**Miscellaneous**											
CLOR	80, 300, 800	95 (12.8)	97 (5.8)	95 (4.9)	96 (14.9)	95 (10.0)	94 (8.4)	10	40.0	101 (11.6)	110 (3.6)

**^a^** WLr = Within-laboratory repeatability while RSD_r_ = Relative standard deviation under repeatability conditions, **^b^** WLR= Within-laboratory reproducibility, while RSD_wR_ = Relative standard deviation under reproducibility conditions **^c^** LOD = Limit of Detection based on S/N = 5, **^d^** LOQ = Limit of Quantitation based on S/N = 10, L1, L2 and L3, refer to each of the three levels at which the validation was performed.

**Table 4:** Preparation of for matrix matched calibration, with corresponding sample concentrations

Spiking Vol. (µL)	Calibration Level	Concentration Ranges (ng L^−1^) for Analyte Group ^a^:									
		A	B	C	D	E	F	G	H	I	J
100	0.5 × L1	20	10	5	0.5	1	0.5	2	2	2.5	2.5
200	L1	40	20	10	1	2	1	4	4	5	5
200	L2	80	40	20	5	5	5	6	6	20	10
200	L3	200	100	40	20	20	20	10	10	40	20
200	L4	300	150	80	50	50	50	14	14	80	50
200	L5	400	200	100	100	100	100	18	18	100	100
200	L6	800	500	200	150	200	200	20	20	200	150
200	L7	1000	1000	500	200	500	500	22	22	500	200
400	L8 (2 × L7)	2000	2000	1000	400	1000	1000	25	25	1000	400

^a^ Analytes within each concentration range group are as described in Section 3.2.

## Supplementary Materials

The following are available online. Figure S1: Structures of anthelmintic compounds, Figure S2: Mean recovery and precision (%RSD, presented as error bars) for assessment of sorbent mass (200 mg vs. 500 mg) and elution volume (10, 15 and 20 mL), Figure S3a: RSM optimiser graph for the 17 analytes selected for assessing the effect of percentage modifier (0–40%) and sample pH (4–10) on extraction, Figure S3b: RSM optimiser graph demonstrating predicted recoveries for the remaining 23 analytes, Figure S4: Increase (green bar) or decrease (red bar) in recoveries for all analytes, when the 20% MeOH sample modifier is incorporated, in comparison to the use of no modifier. The acceptable recovery range is as shown by the upper (120%) and lower (70%) recovery lines, Table S1: Physicochemical data for the studied anthelmintics [40,41] (where available), Table S2: Optimised UHPLC-MS/MS conditions optimised and refined from Whelan et al., 2010 [30], Table S3: Summary of 13 experimental combinations, including 5 center points, generated using MiniTab, for response surface methodology assessing sample modifier and pH.
