# Supplementary material for: Development and Optimisation of a Multiresidue Method for the Determination of 40 Anthelmintic Compounds in Environmental Water Samples by Solid Phase Extraction (SPE) with LC-MS/MS Detection
Source: Molecules. 2019 May 22;24(10):1978. doi: 10.3390/molecules24101978 (PMC6572551; doi:10.3390/molecules24101978)
Supplement: Supplementary file 1 [file molecules-24-01978-s001.pdf]

Supplementary Material

Development and optimisation of a multi-residue method for the determination of 40 anthelmintic compounds in environmental water samples by solid phase extraction (SPE) with LC-MS/MS detection.

Damien Mooney [1, 4, 5, *], Catherine Coxon [1, 5], Karl G Richards [2, 5], Laurence Gill [3, 5], Per-Erik Mellander [2] and Martin Danaher [4]

[1] School of Natural Sciences, Geology Department, Trinity College Dublin, Ireland; mooneyd2@tcd.ie and cecoxon@tcd.ie
[2] Environment, Soils and Land-Use Department, Environment Research Centre, Teagasc, Johnstown Castle, Wexford, Ireland; Karl.Richards@teagasc.ie and PerErik.Mellander@teagasc.ie
[3] Department of Civil, Structural and Environmental Engineering, Trinity College Dublin, Ireland; Laurence.Gill@tcd.ie
[4] Food Safety Department, Teagasc Food Research Centre, Ashtown, Dublin 15, Ireland; damien.t.mooney@teagasc.ie and Martin.Danaher@teagasc.ie
[5] Groundwater spoke, Irish Centre for Research in Applied Geosciences (iCRAG), Ireland
* Correspondence: mooneyd2@tcd.ie (DM) or Martin.Danaher@teagasc.ie (MD); Tel.: +353-1-8059500

Received: date; Accepted: date; Published: date

Contents

Figure S1. Structures of anthelmintic compounds by structural class

- **(a)** structures of benzimidazole anthelmintics
- **(b)** structures of macrocyclic lactone anthelmintics
- **(c)** structures of organophosphate anthelmintics
- **(d)** structures of salicylanilide and substituted phenol anthelmintics
- **(e)** structures of amino-acetonitrile derivative anthelmintics
- **(f)** structures of tetrahydropyrimidines (MOR) and imidazothiazole (LEV) anthelmintics
- **(g)** structure of one miscellaneous anthelmintic (CLOR)

Figure S2. Mean recovery and precision (%RSD, presented as error bars) for assessment of sorbent mass (200 mg vs. 500 mg) each using three elution volume (10, 15 and 20.mL)
Figure S3 (a). RSM optimiser graph for the 17 analytes selected for assessing the effect of percentage modifier (0 – 40%) and sample pH (4 – 10) on extraction
Figure S3 (b). RSM optimiser graph demonstrating predicted recoveries for the remaining 23 analytes, under the selected optimum conditions for percentage modifier and sample pH (20% modifier and pH 7)
Figure S4. Increase (green bar) or decrease (red bar) in recoveries for all analytes, when the 20% MeOH sample modifier is incorporated, in comparison to the use of no modifier

Table S1. Pysicochemical data, where available, for the anthelmintics studied
Table S2. UHPLC-MS/MS conditions optimised and refined from Whelan et al. 2010 [30]
Table S3. Summary of 13 experimental combinations, including 5 center points, generated using MiniTab, for response surface methodology assessing sample modifier (% MeOH) and pH conditions

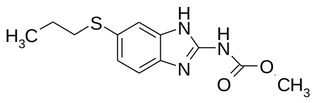

Albendazole

ABZ

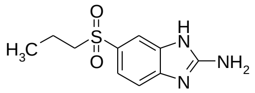

Albendazole amino sulphone

ABZ-NH$_2$-SO$_2$

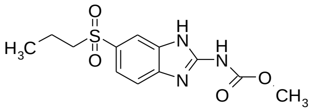

Albendazole sulphone

ABZ-SO$_2$

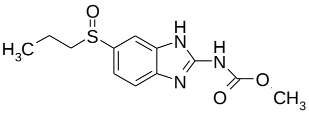

Albendazole sulphoxide

ABZ-SO

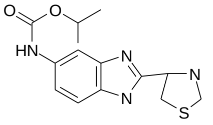

Cambendazole

CAM

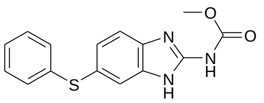

Fenbendazole

FBZ

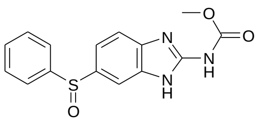

Oxfendazole

OXF

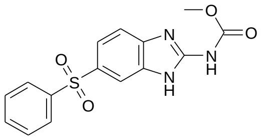

Fenbendazole sulphone

FBZ-SO$_2$

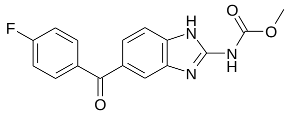

Flubendazole

FLU

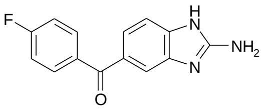

Amino-Flubendazole

FLU-NH$_2$

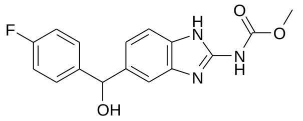

Hydroxy-Flubendazole

FLU-OH

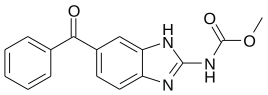

Mebendazole

MBZ

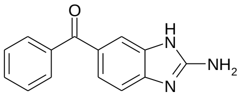

Amino-Mebendazole

MBZ-NH$_2$

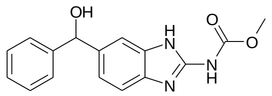

Hydroxy-Mebendazole

MBZ-OH

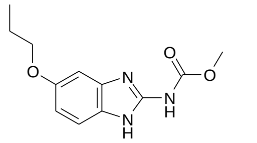

Oxibendazole

OXI

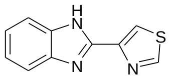

Thiabendazole

TBZ

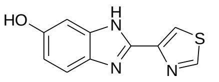

5-Hydroxy Thiabendazole

TBZ-OH

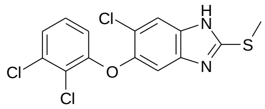

Triclabendazole

TCB

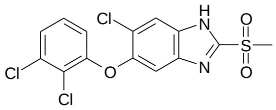

Triclabendazole sulphone

TCB-SO$_2$

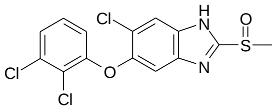

Triclabendazole sulphoxide

TCB-SO

Figure S1 (a) structures of benzimidazole anthelmintics

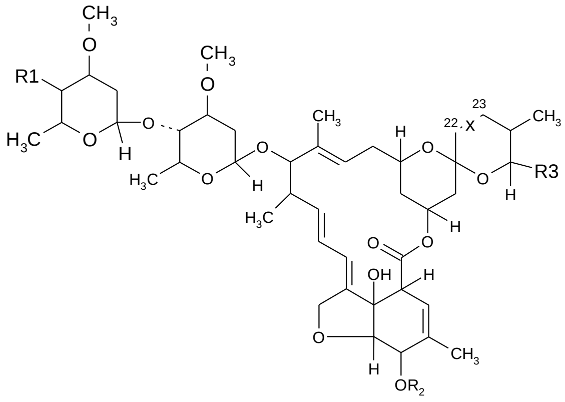

Avermectin	R1	R2	R3	C_{22}-x-CC_{23}
**Abamectin B1a** (ABA)	OH	H	$CHCH_3CH_2CH_3$	-CH=CH-
**Doramectin** (*DORA*)	OH	H	C_6H_{11}	-CH=CH-
**Emamectin Benzoate B1a** (*EMA*)	$C_6H_5COOHCH_3NH$	H	$CHCH_3CH_2CH_3$	-CH=CH-
**Eprinomectin B1a** (*EPRINO*)	$NHCOCH_3$	H	$CHCH_3CH_2CH_3$	-CH=CH-
**Ivermectin B1a** (*IVER*)	OH	H	$CHCH_3CH_2CH_3$	$-CH_2-CH-$

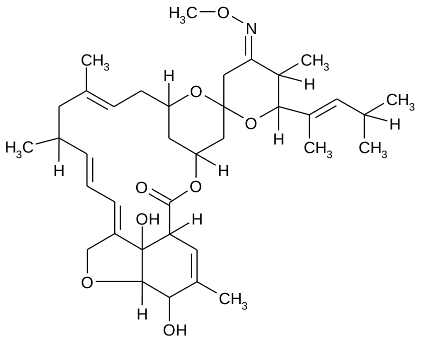

Moxidectin

MOXI

Figure S1 (b) structures of macrocyclic lactone anthelmintics adapted from Tuck et al. [1]

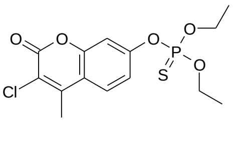

Coumaphos

COUMA

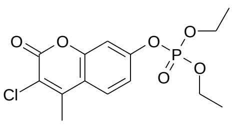

Coumaphos-oxon

COUMA-O

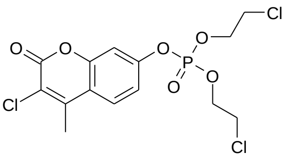

Haloxon

HALOX

Figure S1 (c) structures of organophosphate anthelmintics

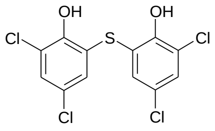

Bithionol

BITH

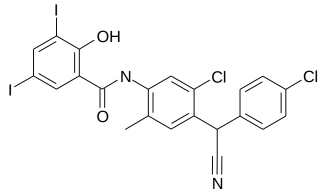

Closantel

CLOS

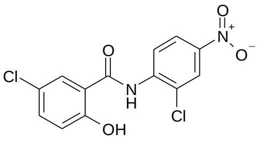

Niclosamide

NICLOS

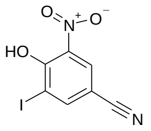

Nitroxynil

NITROX

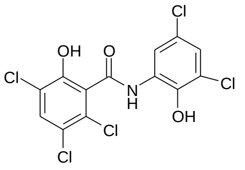

Oxyclozanide

OXY

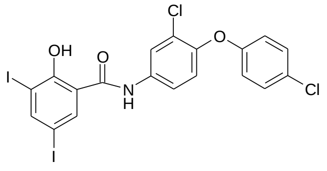

Rafoxanide

RAFOX

Figure S1 (d) structures of salicylanilide and substituted phenol anthelmintics

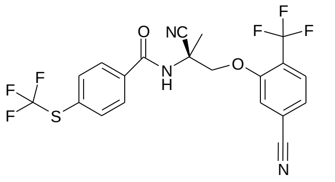

Monepantel

MONE

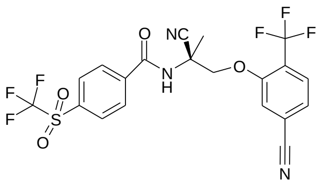

Monepantel sulphone

MONE-SO_2

Figure S1 (e) structures of amino-acetonitrile derivative anthelmintics

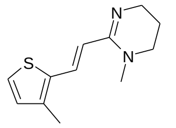

Morantel

MOR

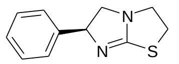

Levamisole

LEV

Figure S1 (f) structures of tetrahydropyrimidines (MOR) and imidazothiazole (LEV) anthelmintics

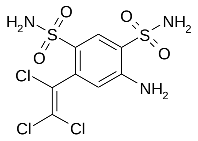

Clorsulon

CLOR

Figure S1 (g) structure of one miscellaneous anthelmintic (CLOR)

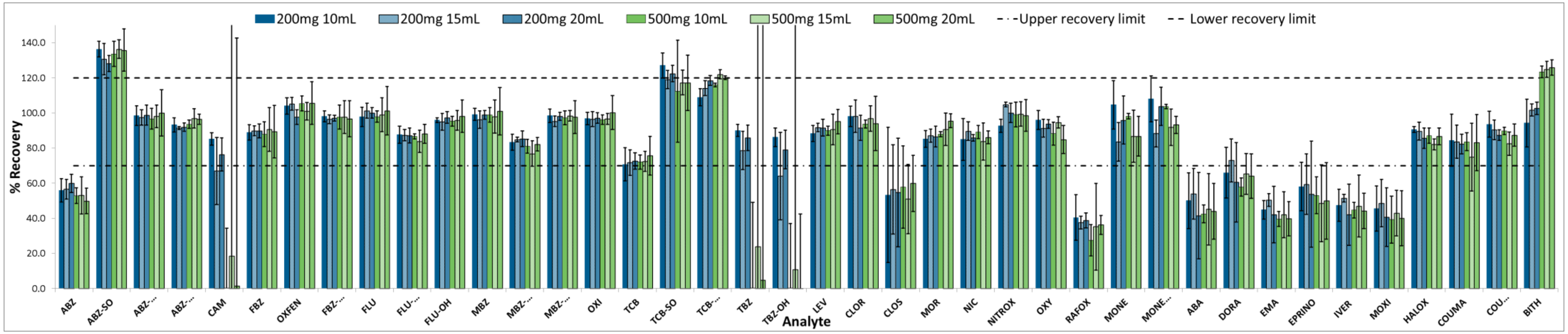

Figure S2: Mean recovery and precision (%RSD, presented as error bars) for assessment of sorbent mass (200 mg vs. 500 mg) each using three elution volume (10, 15 and 20.mL)

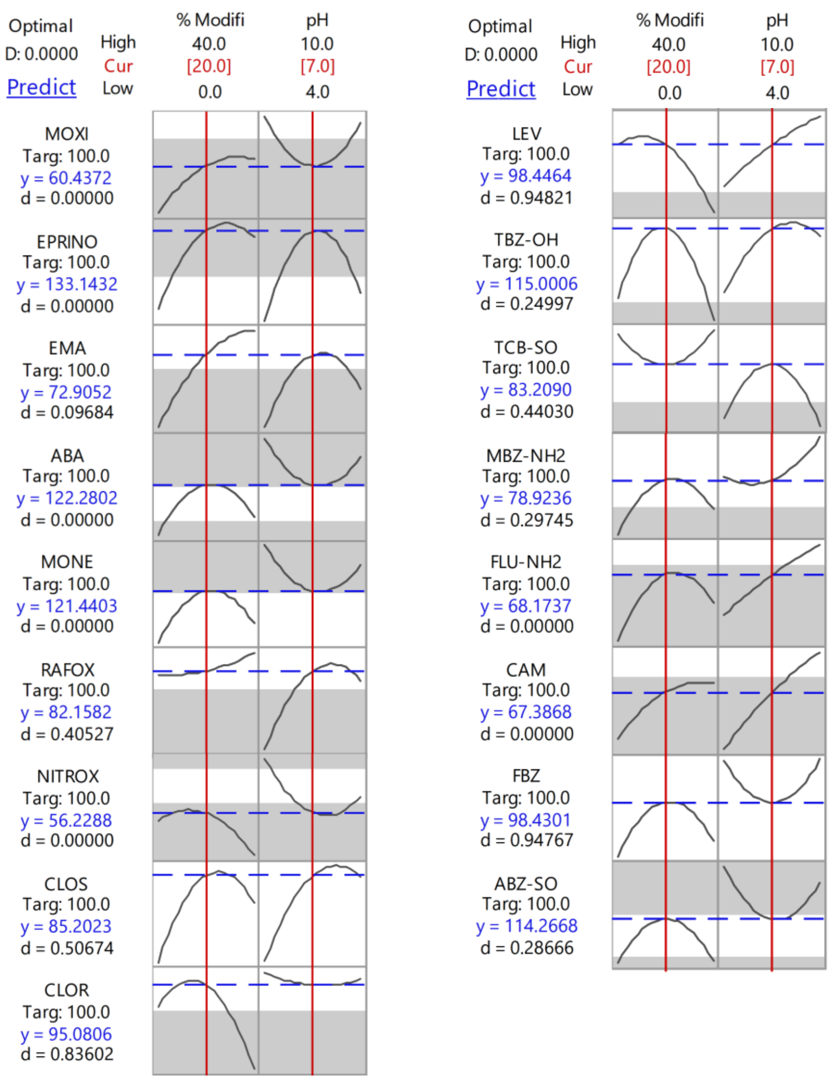

Figure S3(a): RSM optimiser graph for the 17 analytes selected for assessing the effect of percentage modifier (0 – 40%) and sample pH (4 – 10) on extraction

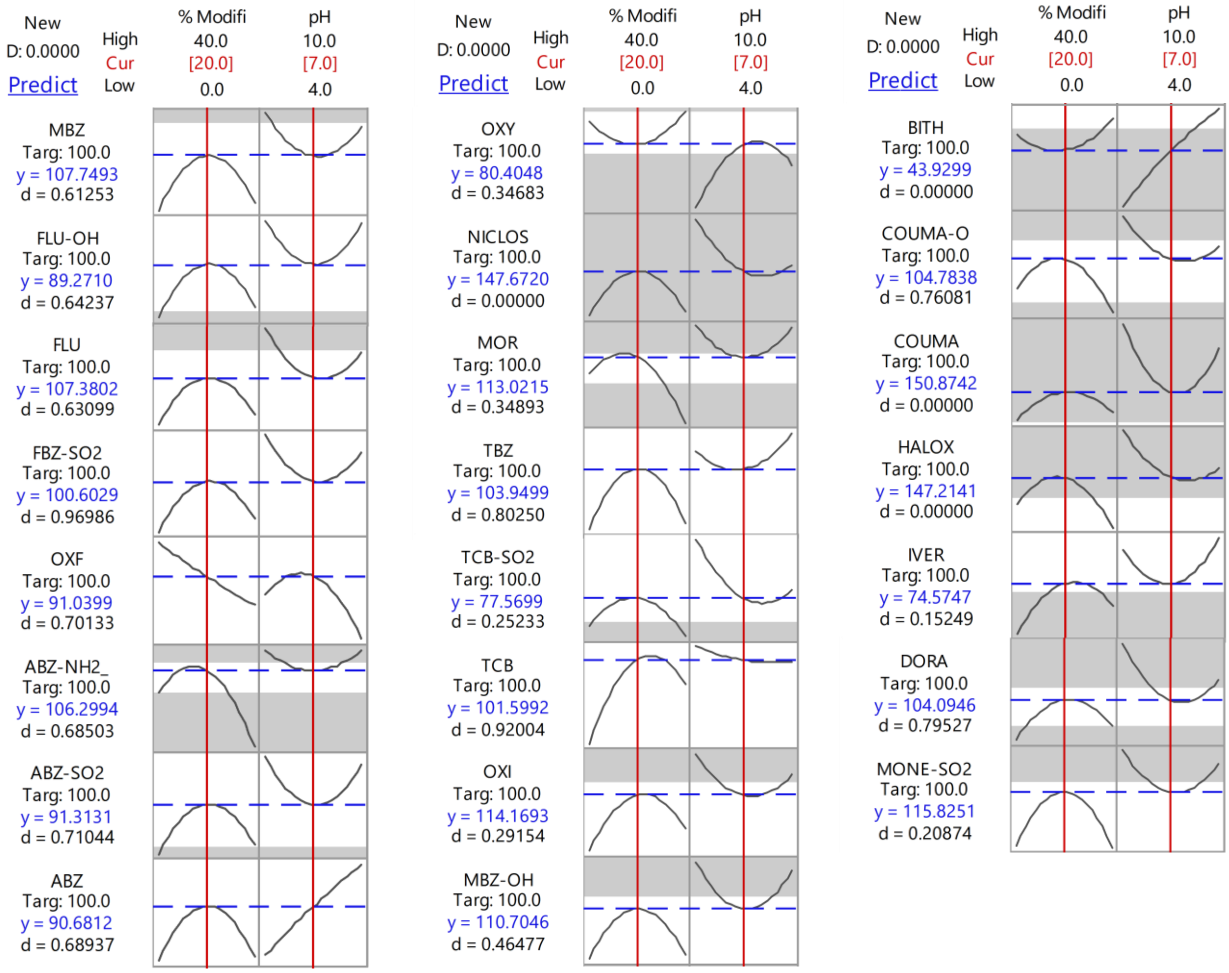

Figure S3(b): RSM optimiser graph demonstrating predicted recoveries for the remaining 23 analytes, under the selected optimum conditions for percentage modifier and sample pH (20% modifier and pH 7)

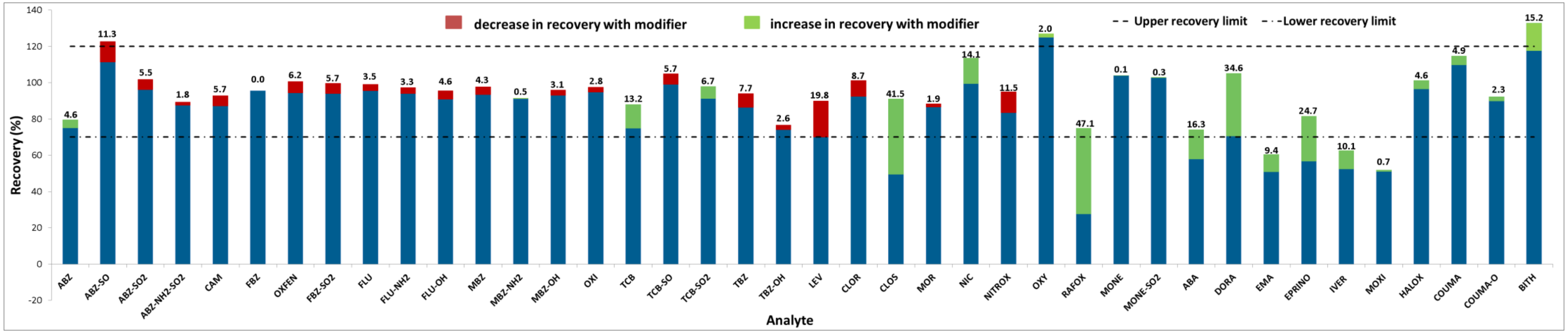

Figure S4. Increase (green bar) or decrease (red bar) in recoveries for all analytes, when the 20% MeOH sample modifier is incorporated, in comparison to the use of no modifier (using HL-DVB SPE cartridge (200mg, 6mL)). The acceptable recovery range is as shown by the upper (120%) and lower (70%) recovery lines.

Table S1 Physicochemical data for the studied anthelmintics (where available)

Class<br>Analyte	P/ TP	S_w<br>mg L^{-1}	logK_{ow}	logK_{oc}	pK_a
**Benzimidazole**					
Albendazole	P	10 [c], 46.39 [f]	3.07 [c] ,<br>2.2-2.92 [e f]	2.94 [c]	3.37, 9.93 [c]<br>5.54, 13.11[f]
Albendazole-sulphoxide	TP	62 [c]	1.2 [c],<br>0.83-0.94 [e]	-	3.5 [c], 9.8 [c]<br>7.8 [e]
Albendazole-sulphone	TP	-	0.9-1.01 [e]	-	-
Albendazole-amino-sulphone	TP	-	0.69-0.75 [e]	-	-
Cambendazole	P				
Fenbendazole	P	0.01-0.04[c],<br>6.38 [f]	1.95 [e] ,<br>3.07-4.01 [e f]	3.37 [c]	5.12, 12.72 [f]
Oxfendazole	TP	407.2 [c f]	1.63[c],<br>1.88-2.13 [e f]	-	4.13, 11.79 [f]
Fenbendazole-sulphone	TP	-	2.13-3.30	-	-
Flubendazole	P	194.3 [c]	2.91 [c],<br>1.98-2.41[e]	3.05 [b c]	3.6, 9.9 [c]
Hydroxy-flubendazole	TP				
Amino-flubendazole	TP				
Mebendazole	P	10 [c], 50.05 [f]	2.71 [c],<br>2.44-2.52 [e]	3.00 [c]	3.5 [e], 9.2 [g]
Hydroxy-mebendazole	TP	-	2.22-2.61 [e]	-	9.8 [e]
Amino-mebendazole	TP	-	1.84-2.27[e]	-	5.5 [e]
Oxibendazole	P	-	1.86-2.63 [e]	-	4.6, 9.6 [g]
Triclabendazole	P	-	5.44 [b c] ,<br>4.90- 6.66 [e]	-	2.5, 10.5 [e],<br>4.6 [g]
Triclabendazole-sulphoxide	TP		3.39-3.66 [e]	-	-
Triclabendazole-sulphone	TP	-	3.58-5.14 [e]	-	-
Thiabendazole	P	335.2 [b c f]	2.47 [c],<br>5.3-6.2 [e]<br>1.58-1.76 [e f]	2.69 [c]	2.5, 4.7 [e] ,<br>5.22, 12.83 [f]
5-Hydroxy-thiabendazole	TP	30 [c]	1.29-1.37 [e]	-	4.5 [e]
**Macro-cyclic lactones (Avermectins and Milbemycins)**					
Abamectin	P	3.5 X 10^{-4} [b c]	4.0 [d]	3.72 – 4.48 [d]	-
Doramectin	P	-	4.1 [d]	3.88-4.94 [d]	-
Emamectin	P	-	5.0 [d]	4.39-5.86 [d]	7.6 [d]<br>4,2, 7.7[h]
Eprinomectin (Benzoate)	P	-	5.40 [d]	3.51-3.96 [d]	-
Ivermectin	P	-	3.22 [d]	3.60-4.41 [d]	-
Moxidectin	P	4 [a c]	4.77 [d], 5.67 [g]	3.90 [b c],<br>4.27-4.63 [d]<br>2.8, 12.6 [g]	-

Class<br>Analyte	P/ TP	S_w<br>mg L^{-1}	logK_{ow}	logK_{oc}	pK_a
**Salicylanilides and substituted phenols**					
Bithionol	P	0.2 [b c]	5.91 [b c]	4.67 [c]	4.83, 10.50 [c]
Closantel	P	1.5×10^{-5} [c]	8.11 [b c]	5.72 [c]	-
Niclosamide	P	10 [a c]	4.56 [b c]	3.58 [c]	-
Nitroxynil	P				
Oxyclozanide	P				
Rafoxanide	P	4.6×10^{-5} [b c]	8.14 [b c]	5.40 [c]	-
**Tetrahydropyrimidines**					
Morantel	P	1.5×10^{5} [a c]<br>(tartrate)	3.69 [c]	2.9 [c]	
**Imidazothiazoles**					
Levamisole	P	1116 [c]<br>$1x\ 10^{5}$ (HCl)	2.87 [b c]	1.88 [c]	-
**Organophosphates**					
Coumaphos	P	-	-	-	-
Coumaphos Oxon	P	-	-	-	-
Haloxon	P	-	-	-	-
**Amino-acetonitrile derivatives**					
Monepantel	P	-	-	-	-
Monepantel-sulphone	TP	-	-	-	-
**Miscellaneous**					
Clorsulon	P	-	-	-	-

(a) extracted from the Merck Index [40]

(b) calculated values using EPI Suite software (EPI WEB 4.0), 2009

(c) extracted and adapted from Horvat *et al.* [13] Table 2.

(d) extracted and adapted from Krogh *et al.* [27] Table 1

(e) extracted and adapted from Danaher et al [4] Table 1

(f) extracted and adapted from santaladchaiyakit *et al.* [29]

(g) extracted and adapted from Zrncic et al [26] Table 1

(h) extracted from van der Velde-Koerts [41]

P = parent compounds, TP = transformation product, S_W = water solubility, logK_{OW} = octanol water partition coefficient, logK_{OC} = soil organic carbon – water partitioning coefficient, pKa = acid dissociation constant

Table S2: UHPLC-MS/MS conditions optimised and refined from Whelan et al. 2010 [30]

Analyte	t_R (min)	MRM/F	M	Pre-ion (m/z)	Product Ions (*m/z*)	Dwell (s)	C (V)	CE (V)	IS
ABZ-NH_2SO_2-d_3	1.54	1	+	242.90	132.95	0.100	40	30	-
LEVA-d_5	1.55	1	+	210.10	183.05	0.100	40	21	-
ABZ-NH_2SO_2	1.57	1	+	240.05	**133.00**/198.00	0.100	40	26/18	ABZ-NH_2-SO_2-d_3
LEVA	1.58	1	+	205.10	122.90/**177.91**	0.100	40	28/20	LEVA-d_5
5-OH-TBZ	1.60	1	+	217.87	146.87/**190.85**	0.100	40	32/26	ABZ- NH_2-SO_2-d_3
TBZ-$^{13}C_6$	3.02	2	+	208.00	181.00	0.025	45	25	-
TBZ	3.03	2	+	201.90	130.90/**174.90**	0.025	45	32/25	TBZ-$^{13}C_6$
ABZ-SO-d_3	3.09	2	+	285.20	243.02	0.010	25	12	-
ABZ-SO	3.11	2	+	282.30	158.95/**240.00**	0.010	25	38/13	ABZ-SO-d_3
MBZ-NH_2	3.26	2	+	238.10	**104.90**/132.90	0.010	45	27/35	TCB-NH_2 (pos)
ABZ SO_2-d_3	3.42	2	+	301.00	158.95	0.010	35	38	-
ABZ-SO_2	3.44	2	+	298.20	**158.90**/266.00	0.010	35	35/20	ABZ-SO_2-d_3
FLU-NH_2	3.56	2	+	256.06	94.90/**122.95**	0.010	45	37/28	TCB-NH_2 (pos)
MOR	2.58,2.95	2	+	221.05	110.90/**122.90**	0.075	35	23/34	TBZ-$^{13}C_6$
NITROX	2.84	3	-	289.00	**126.85**/161.90	0.006	40	24/20	NITROX-$^{13}C_6$
NITROX-$^{13}C_6$	2.89	3	-	295.00	126.69	0.006	40	25	-
CLOR	3.10	3	-	379.90	**343.80**	0.006	18	12	SAL
	"	"	"	377.90	341.80	"	"	12	"
FBZ-SO-d_3	3.92	4	+	321.04	158.95	0.005	35	32	-
OXF	3.93	4	+	316.10	**159.05**, 191.09	0.005	35	30/24	FBZ-SO-d_3
MBZ-OH-d_3	4.07	4	+	301.15	16.05	0.008	35	32	-
MBZ-OH	4.09	4	+	298.25	160.05/**266.15**	0.010	35	36/22	MBZ-OH-d_3
FBZ-SO_2-d_3	4.27	4	+	335.05	299.90	0.010	35	23	-
FBZ-SO_2	4.28	4	+	331.9	158.90, **300.00**	0.010	35	36/21	FBZ-SO_2-d_3
FLU-OH	4.37	4	+	316.2	125.10, **160.05**	0.010	40	33/35	MBZ-OH-d_3
CAM	4.58	4	+	302.96	216.85, **260.95**	0.008	35	26/18	FBZ-d_3
TFM	4.55	5	-	205.95	159.95	0.051	35	24	-
SAL	5.47	5	-	212.05	92.00	0.021	35	28	-

Analyte	t_R (min)	MRM/F	M	Pre-ion (m/z)	Product Ions (*m*/*z*)	Dwell (s)	C (V)	CE (V)	IS
OXI-d_7	4.80	6	+	257.15	177.05	0.006	35	28	-
OXI	4.86	6	+	249.90	175.90/**218.00**	0.006	35	26/18	OXI-d_7
MBZ-d_3	5.00	6	+	299.15	105.05	0.006	40	33	-
MBZ	5.02	6	+	296.14	105.05/**264.10**	0.006	35	32/18	MBZ-d_3
FLU-d_3	5.24	6	+	318.15	123.00	0.006	40	36	-
FLU	5.26	6	+	313.80	123.00/**282.00**	0.006	40	35/24	FLU-d_3
ABZ-d_3	5.69	6	+	269.12	233.85	0.006	35	19	-
ABZ	5.70	6	+	266.07	**191.03**/234.00	0.006	35	32/13	ABZ-d_3
COUMA-O	5.93	7	+	347.01	**210.99**/291.20	0.005	30	29/22	FBZ-d_3
HALOX	6.08	7	+	414.90	**211.00**/272.95	0.005	35	35/32	ABZ-d_3
FBZ-d_3	6.12	7	+	303.00	267.95	0.005	35	22	-
FBZ	6.13	7	+	300.01	159.01/**268.01**	0.005	35	24/23	FBZ-d_3
TCB NH_2 (pos)	6.25	7	+	328.00	166.95	0.005	48	27	-
COUMA	6.80	8	+	363.02	227.05/**307.05**	0.008	35	25/16	ABZ-d_3
TCB	6.87	8	+	359.04	**274.07**/343.97	0.008	45	36/27	TCB-d_3
TCB-d_3	6.87	8	+	361.90	343.90	0.008	45	25	-
TCB-SO_2	6.12	9	-	389.00	244.16/**309.94**	0.006	55	38/35	TCB-NH_2(neg)
TCB-NH_2 (neg)	6.25	9	-	325.87	180.90	0.006	45	26	-
MONE-SO_2	6.50	9	-	504.00	165.85/**185.94**	0.006	15	50/15	CLOS-$^{13}C_6$
OXY	6.52	9	-	397.95	**176.00**/201.90	0.006	30	28/23	OXY-$^{13}C_6$
OXY-$^{13}C_6$	6.52	9	-	403.75	175.90	0.006	30	23	-
TCB SO	6.56	9	-	375.00	181.00/**213.00**	0.006	30	27/35	TCB-NH_2(neg)
MONE	6.72	9	-	472.00	166.00/**185.91**	0.006	15	45/13	CLOS-$^{13}C_6$
NICLOS	6.77	9	-	324.95	**170.91**/288.89	0.006	35	26/17	SAL
BITH	6.99	10	-	352.90	**160.95**/191.95	0.005	28	27/22	RAFOX-$^{13}C_6$
CLOS	7.01	10	-	660.85	**126.90**/315.10	0.015	40	43/35	CLOS-$^{13}C_6$
CLOS-$^{13}C_6$	7.01	10	-	666.85	126.94	0.015	50	45	-
RAFOX	7.20	10	-	623.79	**126.87**/344.83	0.015	50	48/31	RAFOX-$^{13}C_6$
RAFOX-$^{13}C_6$	7.21	10	-	630.95	126.99	0.015	50	40	-
EMA	7.43	11	+	886.65	126.10/**158.10**	0.005	40	38/37	SEL

Analyte	t_R (min)	MRM/F	M	Pre-ion (m/z)	Product Ions (*m/z*)	Dwell (s)	C (V)	CE (V)	IS
EPRINO	7.64	11	+	915.55	**144.15**/298.15	0.015	15	41/8	SEL
ABA	7.74	11	+	890.40	**305.15**/567.00	0.005	15	25/13	SEL
MOXI	7.92	11	+	640.30	498.10/**528.00**	0.005	15	11/9	SEL
DORA	7.94	11	+	916.60	**331.10**/593.10	0.005	15	25/13	SEL
SEL	8.14	11	+	770.40	333.30	0.005	40	22	-
IVER	8.21	11	+	892.40	**307.15**/569.10	0.005	20	26/14	SEL

t_R= Retention time, MRM/F = MRM window function where (1) 1.10 - 2.60 min (2) 1.90 - 3.90min (3)2.60 - 3.60min (4) 3.70 - 5.10min (5) 4.70 - 5.90 min (6) 4.50 - 6.00 min (7) 5.80 - 6.60min (8) 6.60 - 7.05min (9) 6.00 - 7.00min (10) 6.90 - 7.60min (11) 7.20 - 8.70min., M = ESI polarity mode; (+) = positive mode and (-) = negative mode , C= cone voltage, CE= collision energy, IS= internal standard. Product ion: quantifier shown in **bold**

Table S3: Summary of 13 experimental combinations, including 5 center points, generated using MiniTab, for response surface methodology assessing sample modifier (% MeOH) and pH conditions

Experiment	Modifier (%)	pH
1	20	7.0
2	20	7.0
3	20	7.0
4	0	4.0
5	20	5.5
6	20	7.0
7	40	10.0
8	20	7.0
9	0	10.0
10	20	8.5
11	40	4.0
12	10	7.0
13	30	7.0
